# Computational evaluation of TIS annotation for prokaryotic genomes

**DOI:** 10.1186/1471-2105-9-160

**Published:** 2008-03-25

**Authors:** Gang-Qing Hu, Xiaobin Zheng, Li-Ning Ju, Huaiqiu Zhu, Zhen-Su She

**Affiliations:** 1 State Key Lab for Turbulence and Complex System and Department of Biomedical Engineering, College of Engineering, Peking University, Beijing 100871, China; 2 Center for Theoretical Biology, Peking University, Beijing 100871, China; 3 Department of Mathematics, University of California, Los Angeles, Los Angeles, CA 90095, USA

## Abstract

**Background:**

Accurate annotation of translation initiation sites (TISs) is essential for understanding the translation initiation mechanism. However, the reliability of TIS annotation in widely used databases such as RefSeq is uncertain due to the lack of experimental benchmarks.

**Results:**

Based on a homogeneity assumption that gene translation-related signals are uniformly distributed across a genome, we have established a computational method for a large-scale quantitative assessment of the reliability of TIS annotations for any prokaryotic genome. The method consists of modeling a positional weight matrix (PWM) of aligned sequences around predicted TISs in terms of a linear combination of three elementary PWMs, one for true TIS and the two others for false TISs. The three elementary PWMs are obtained using a reference set with highly reliable TIS predictions. A generalized least square estimator determines the weighting of the true TIS in the observed PWM, from which the accuracy of the prediction is derived. The validity of the method and the extent of the limitation of the assumptions are explicitly addressed by testing on experimentally verified TISs with variable accuracy of the reference sets. The method is applied to estimate the accuracy of TIS annotations that are provided on public databases such as RefSeq and ProTISA and by programs such as EasyGene, GeneMarkS, Glimmer 3 and TiCo. It is shown that RefSeq's TIS prediction is significantly less accurate than two recent predictors, Tico and ProTISA. With convincing proofs, we show two general preferential biases in the RefSeq annotation, *i.e*. over-annotating the longest open reading frame (LORF) and under-annotating ATG start codon. Finally, we have established a new TIS database, SupTISA, based on the best prediction of all the predictors; SupTISA has achieved an average accuracy of 92% over all 532 complete genomes.

**Conclusion:**

Large-scale computational evaluation of TIS annotation has been achieved. A new TIS database much better than RefSeq has been constructed, and it provides a valuable resource for further TIS studies.

## Background

To initiate translation in prokaryote, a ribosome binds to a specific region of mRNA and then recognizes a nearby start codon. The position of the first nucleotide base pair (bp) in the start codon is denoted by translation initiation site (TIS). The sequence upstream to the TIS, the start codon itself and the sequence downstream to the TIS show specific patterns which differ from genome to genome. The sequence at about 20 bps upstream to the TIS in most prokaryotic genes contains primarily purine rich Shine-Dalgarno sequence [1]. However, increasing numbers of genes with missing Shine-Dalgarno sequences, known as leaderless genes if they also lack a 5'-untranslated region, have been reported in archaeal genomes [2]. Genome-wide computational analysis on leaderless genes revealed A/T rich sequences in a region at about 30 bps further upstream [3]. The start codon in most cases shows a strong preference to the ATG triplet than to others such as TTG and GTG [4]. Sequences downstream to the TIS exhibit a periodicity of three in the codon usage. Comparative genomic studies show that the sequence patterns around the true TIS might differ significantly between genomes. With the aid of a sequence logo tool, Torarinsson *et al*. [3] and Zhu *et al*. [5] reported the variation of sequence patterns among dozens of archaeal genomes, which shed light on the understanding of the divergence of translation initiation mechanisms in prokaryote.

Knowledge of exact TIS is essential for conducting experiments involving the identification of natively purified proteins by N-terminal amino acid sequencing as well as heterologous protein production [6]. However, there are increasing concerns on the TIS annotation quality in widely used databases such as GenBank and RefSeq [5-9]. Earlier completed microbial genome projects tend to annotate the 5'-most candidate start which is in frame to the stop codon [7]. On the other hand, Poole *et al*. [6] has observed a strong discrepancy of TIS annotation between databases CMR and RefSeq on several genomes. Despite manual corrections and periodic updates, the quality of the current TIS annotations is still largely uncertain, and it is intriguing to develop an independent method for assessing the TIS annotation reliability. Such method, if successful, may also be helpful to provide hints for further improvement. The need for developing such method is becoming more urgent for the database such as RefSeq is so widely used by experimental biologists that errors in the annotation might have big impact.

Several attempts have been made to assess the reliability of TIS annotation. Nielsen and Krogh [8] were the first to make a serious large-scale assessment of the reliability of the TIS annotation in RefSeq, but their approach that takes EasyGene 1.2 as the "gold standard" for comparison is questionable. As we will see later, EasyGene's own accuracy is not outstanding, hence the biased assessment is of limited interest. Frishman *et al*. [10], using the Orpheus program, show that the information content of aligned TIS upstream sequences correlates with the TIS prediction accuracy. Zhu, *et al*. [5] made a qualitative assessment of the relative TIS annotation quality for two TIS predictors, by comparing the sequence logo [11] of aligned TIS upstream sequences. In this assessment, the sequence logo around the aligned TISs of a consensus set predicted by both predictors (called consensus logo) is considered to be reliable, and hence the difference to the sequence logo of the aligned TISs of a 'specific' set predicted by only one program (called specific logo) would indicate qualitatively the TIS accuracy of that program. Taking *S. solfataricus *as an example, Zhu, *et al*. [5] showed that the specific sequence logo of MED 2.0 is very similar to the consensus logo obtained jointly with GenBank annotation, but the specific logo of the GenBank shows almost no sequence pattern. This result suggests that the GenBank TIS annotation in *S. solfataricus *is lower than MED 2.0. Generally speaking, there exists no systematic method to computationally evaluate the accuracy of TIS prediction.

We propose here a computational method to quantitatively estimate the TIS annotation accuracy of a prokaryotic genome; the annotation can be provided by either a program or a database. The method is based on a homogeneity assumption that the sequence patterns represented by a PWM around TISs are homogenous for a generic subset of genes of a genome. The whole set of TIS predictions are split into two sets; set I is called reference set and is so constructed to be nearly 100% accurate (see section "Reference set") and set O has only partially accurate prediction which are to be quantitatively evaluated. We assume that the set I and O are generic subsets; this assumption is diffcult to prove, but is sound as a first approximation. It is then assumed that the PWM around predicted TISs in the set O can be modelled as a linear combination of three elementary PWMs, one around true TIS and the others two around false TISs which are located upstream and downstream to the true TIS, respectively. All the three elementary PWMs are obtained from the sequence patterns of the reference set I, which carries naturally genome-specific features. A generalized least square estimator then determines the weighting of each of the three PWMs, and the weighting of the true TIS naturally determines the accuracy of the TIS annotation in the set O. Hence, the prediction accuracy over the entire genome, I ⋃ O, is derived.

The validity of the method is established with tests on experimentally verified TISs set EcoGene [12]. Then, the method is applied to estimate the TIS annotation accuracy of 532 genomes on the public databases and publicly available programs such as RefSeq [13], ProTISA [14], EasyGene [8,15], GeneMarkS [7], Glimmer 3 [16] and TiCo [17]. Finally, this analysis has led to a construction of a new TIS database, SupTISA, which is much better than RefSeq on TIS annotations.

## Methods

### Basic definitions

Let us first introduce several definitions:

• a blackboard bold symbol X denotes a set of genes with specified STOP and TIS;

• the sample size of X is denoted by $\Omega_{X}$;

• the symbol $A_{X}$ denotes the accuracy of X;

• the symbol S denotes the set of annotation;

• the symbol I denotes the reference set whose TISs are supposed to be 100% accurate, and the symbol O denotes its compliment: S=I∪O;

• the symbol T denotes a subset of O which has correct TIS annotation, and the symbol F denotes its compliment: O=T∪F. Thus the annotation accuracy of O can be expressed as $A_{O}≜\Omega_{T}/\Omega_{O}$. Furthermore, the overall annotation accuracy is given by

(1) $$A_{S}≜\frac{\Omega_{I}}{\Omega_{S}}+\frac{\Omega_{O}}{\Omega_{S}}A_{O}.$$

### Elementary patterns expressed with PWMs

The main task of this work is to estimate $A_{O}$. The tool for this evaluation is the PWM of aligned sequences around TIS. We choose *l *bps upstream and *r *bps downstream of start codons (in this paper *l *= 50 and *r *= 15) to form a window of width *l *+ *r*. The PWM for the set X is denoted by $W_{X}$; concretely, the frequency of nucleotide *b *at an aligned position *j *is denoted by *W*_*j *_(*b*), where *b *= 1 denotes adenine (A), *b *= 2 denotes cytosine (C), and so forth.

Three elementary PWMs will be relevant to our analysis, and correspond to three types of TISs in the annotation. The first is true TIS, and the corresponding PWM is denoted by $W_{T}$. The second and third are two types of false TIS, whose PWMs will be denoted by $W_{F_{u}}$ and $W_{F_{d}}$ indicating the false TIS located either upstream or downstream of true TIS, respectively. Note that the overall PWM is, by definition, a linear combination of PWMs of sub-patterns, and this linearity has a consequence that any number of sub-patterns around true TISs can always be combined to be a single $W_{T}$, and this is also a valid statement for $W_{F_{u}}$ and $W_{F_{d}}$ As long as the distribution of sub-patterns are uniform for the set I and O (which is our homogeneity assumption, see below), it is justified to use the three elementary PWMs to represent an actual observed PWM such as $W_{O}$.

**Figure 1:**
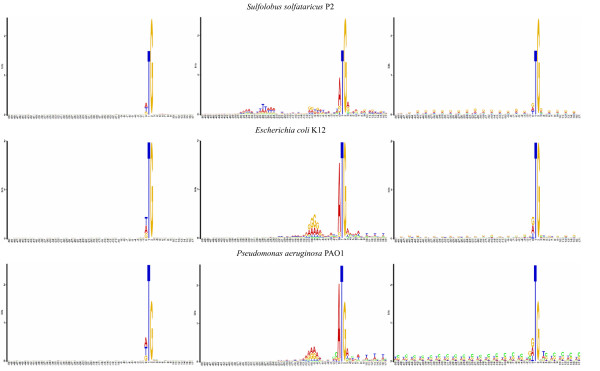
**Three elementary sequence patterns around TISs**. We selected three genomes with widely different genomic GC content to illustrate the content of three elementary PWMs associated with different TISs. Three genomes are from top to bottom: *Sulfolobus solfataricus *P2 (35.8%), *Escherichia coli *K12 (50.8%), and *Pseudomonas aeruginosa *PAO1 (66.6%). Logos from the left to right correspond to $W_{F_{d}}$, $W_{T}$ and $W_{F_{u}}$ respectively. Data are obtained from the reference set and logos are generated by [11].

The difference between the three types of PWMs are biologically clear. $W_{T}$ contains regulatory signals such as the SD sequence, which are required by the translation initiation machinery. Evolution must conserve such pattern. On the other hand, $W_{F_{u}}$ characterizes sequences exposed to neutral evolution and hence is generally feature-less. Finally, a false TIS located downstream to the true TIS is surrounded by coding sequences and $W_{F_{d}}$ exhibits period three oscillations. In Figure 1, we show the three patterns, obtained by our study, for three different organisms. The features discussed above are generally present.

An annotation of finite accuracy will give rise to a PWM which is a linear combination of the above three PWMs. Specifically, for the set O, we write:

(2) $$W_{O}=\alpha_{T}W_{T}+\alpha_{F_{u}}W_{F_{u}}+\alpha_{F_{d}}W_{F_{d}},$$

where $\alpha_{T}+\alpha_{F_{u}}+\alpha_{F_{d}}=1$. We will develop a least square estimator to determine the three coeffcients *α'*s from the above four observed PWMs, the first coming from the set O and the last three from the set I.

### Reference set

The three elementary PWMs are obtained from the reference set, which is very important in this evaluation. The reference set needs to be as reliable as possible, and should not be biased towards any database/predictor to be evaluated. We have chosen to use the six most recent TIS databases/predictors, namely, RefSeq [13], ProTISA [14], EasyGene [8,15], GeneMarkS [7], Glimmer 3 [16] and TiCo [17], to derive the reference set. For any genome, we obtained the reference TIS set by intersecting the annotations of all six databases/predictors; in order to reduce false positives, genes less than 600 bps are excluded [18]. Among the six annotations, EasyGene, GeneMarkS, Glimmer 3 and TiCo achieve significant improvements on TIS prediction [7,15-17], and ProTISA is compiled to contain more than 390, 000 confirmed TISs with collected evidence from experiments, literatures, conserved domain search, and sequence alignment between orthologous genes [14]. Today, we can get the intersecting of all 532 genomes found on GenBank from all the databases/predictors except EasyGene, the later only provides annotations of 157 genomes. We will use only five of the six annotations to get the reference set for the remaining 381 genomes. These reference sets represent the best TIS predictions so far achieved, which do not cover all genes (41.5 ± 9.5% in RefSeq) but presumably very accurate. Our present work provide an evaluation for the rest of the predictions, i.e. that of the set O.

The procedure to obtain three PWMs from the reference set is as follows. Since the true TISs are known, the aligned sequences around the true TISs directly give rise to ${\hat{W}}_{T}$ (an estimate of $W_{T}$, see later). Similarly, one obtains ${\hat{W}}_{F_{u}}$ and ${\hat{W}}_{F_{d}}$ by aligning sequences around a randomly chosen false TIS upstream or downstream to the true TIS. Note that ${\hat{W}}_{F_{u}}$ contains the least feature among the three,. Note also that in this procedure, all three PWMs have the sample size: $\Omega_{I}$, so there will be finite size effects to be accounted for in the analysis below.

### Homogeneity assumption

Finally, let us discuss the limitation of the homogeneity assumption. The sequence pattern encompasses regulatory signals which are important to the translation of genes. The homogeneity property is based on the idea that the translation mechanism is largely universal across a genome. There may be several translation mechanisms acting on a genome [2,3,5,14]; in this case, the homogeneity assumption requires that the proportions of the sub-patterns remain the same for different subsets of our interest, namely the set I and O. To prove this is a diffcult problem, and we do not intend to accomplish it in this work. This is because that O might contain TISs with different statistical properties from the reference set, which makes them harder be annotated correctly. However, the validity of our evaluation depends on how large is its effect. A deviation from the homogeneity is similar to the effect of finite accuracy for the set I, which is easier to study. The testing results (see section "Testing") show that imperfection or bias in the set I yields definite but small modification of the evaluated accuracy. Therefore, it is reasonable to conclude that the homogeneity assumption is sound to leading order and the results of our evaluation are believable.

### Algorithm

Let ${\hat{W}}_{X}$ be an estimate of $W_{X}$. Because we are disposed with a finite set of samples, Eq. 2 becomes

(3) $$W_{O}=\alpha_{T}{\hat{W}}_{T}+\alpha_{F_{u}}{\hat{W}}_{F_{u}}+\alpha_{F_{d}}{\hat{W}}_{F_{d}}+\varepsilon ,$$

where *ε *depends on both $\Omega_{O}$ (for $W_{O}$) and $\Omega_{I}$ (for the three elementary *W'*s). Furthermore, to eliminate redundancy from data, it is wise to make a Z-transformation [19] from the matrix *W *of (*l *+ *r*) × 4 dimensions to a matrix *V *of (*l *+ *r*) × 3 dimensions:

(4) $$\left\{ \begin{array}{l} V_{j}(1)=W_{j}(1)+W_{j}(2)-W_{j}(3)-W_{j}(4)\\ V_{j}(2)=W_{j}(1)-W_{j}(2)+W_{j}(3)-W_{j}(4)\\ V_{j}(3)=W_{j}(1)-W_{j}(2)-W_{j}(3)+W_{j}(4) \end{array}\right.$$

where *j *= 1, 2,..., *l*+*r*. Consequently, we rewrite Eq. 3 as

(5) $$V_{O}=\alpha_{T}{\hat{V}}_{T}+\alpha_{F_{u}}{\hat{V}}_{F_{u}}+\alpha_{F_{d}}{\hat{V}}_{F_{d}}+\varepsilon^{\prime }.$$

The nucleotide frequencies at different positions in all the PWMs are assumed to be independent [20]. The assumption is widely applied in gene-finders [5,7,16], and deviations are expected to be small based on results presented in the "testing" section.

Together with the homogeneity assumption, we show that *E*(*ε'*) = 0 and

(6) $$Var(\varepsilon^{\prime })=\sum_{X}\left((\frac{\alpha_{X}^{2}}{\Omega_{I}}+\frac{\alpha_{X}}{\Omega_{O}}){\Sigma^{\prime }}_{X}\right)$$

where X takes T, $F_{u}$ and $F_{d}$, respectively, and ${\Sigma^{\prime }}_{X}$ is a 3(*l*+*r*) × 3(*l*+*r*) covariance matrix calculated on the set X whose components are inferred from ${\hat{W}}_{X}$ (see Additional File 1).

The estimation of $\alpha_{X}$s in Eq. 6 can be done using a generalized least square, namely by minimizing the following weighted sum of squared errors *t *(see Additional File 1):

(7) $$\arg\underset{\alpha }{\min}\{Err\}=\arg\underset{\alpha }{\min}\{{\varepsilon^{\prime }}^{T}{\Sigma^{\prime }}^{-1}\varepsilon^{\prime }\}$$

where *α *denotes the vector ($\alpha_{T}$, $\alpha_{F_{u}}$, $\alpha_{F_{d}}$)^*T *^and Σ ^' ^denotes *Var*(*ε'*) for simplification. Because of Eq. 6, Σ' has a complicate dependence on *α*, and we need to solve a nonlinear optimization problem. This is done by an iterative procedure, with an initial *α *to evaluate Σ' which is substituted into a group of linear equations of optimization (the first-order partial derivative of *α *equals to zero) to calculate new *α*. The new *α *is then used to update Σ', and the calculation repeats until *α *converges. As explained in details in Additional File 1, the calculations converge quickly to correct values. Throughout our tests, we did not encounter any instability for this calculation.

Throughout the calculation, we face a question of how reliable the estimates of $W_{T}$, $W_{F_{d}}$, $W_{F_{u}}$ and $W_{O}$ are, given the finite sample of gene sequences used for the evaluation. This problem is addressed by adopting a bootstrapping strategy for finding a confidence interval (CI) of $\alpha_{T}$. The calculation is repeated 200 times; each time, we randomly select, with replacement, a sample of TISs from the reference set of size $\Omega_{I}$ to calculate three elementary $\hat{W}$s and a sample of TISs from the set O of size $\Omega_{O}$ to calculate ${\hat{W}}_{O}$, and perform the optimization calculation described above. This calculation is carried out during the testing and every assessment. The consistency of the estimate is then judged by the uncertainty interval of the output accuracy.

## Results

### Testing

The experimentally confirmed TISs in EcoGene [12], denoted as *EcoGene854*, allows us to design a procedure to test the reliability of our method. The procedure goes as follows. First, randomly divide genes in *EcoGene854 *into two equal-size-set O and I, and calculate the three elementary PWMs from the set I, as explained above. Then, we create a series of partially accurate O with accuracy *α *from 40% to 90% at a step of 10% by replacing 100(1 -*α*)% of the true TISs by randomly choosing false TISs. The aligned sequences with the newly assigned TISs of the set O gives rise to $W_{O}$, which is a simulated real annotation PWM of finite accuracy. The generalized least square calculation determines the estimated accuracy, $\hat{\alpha }$. For each *α*, we repeat the generation of the set I and O (200 times) and obtain a distribution of $\hat{\alpha }$ from which the average and standard deviation of $\hat{\alpha }$ can be derived.

**Figure 2:**
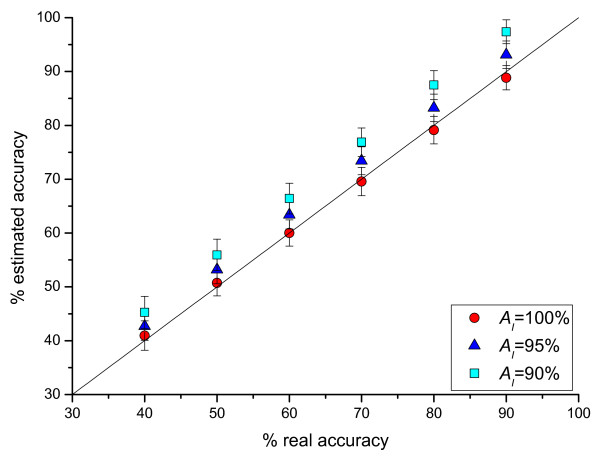
Comparison of estimated versus exact accuracy at three reference sets of different accuracy.

In Figure 2, we plot the average estimate and the standard deviation of $\hat{\alpha }$ as a function of true accuracy *α*. When the reference set is 100% accurate, $\hat{\alpha }$ fluctuates around *α *with ± 2.6%. We have also found that the estimate $\hat{\alpha }$ is unbiased.

An intriguing question is what happens if the reference set is not 100% accurate. This can be easily checked by carrying out a series of tests with varying accuracy of I (by randomly replacing a portion of true TISs by false ones): $A_{I}$ = 90%, 95% and 100%. The results are also shown in Figure 2. Generally speaking, an over-estimation of the accuracy is obtained. This is readily understood because when $A_{I}$ < 100%, $W_{T}$ contains contribution from false TIS, and hence a bias is generated in favor of false annotation, and the estimated accuracy is higher. Specifically, we found that the estimated accuracy is about $\hat{\alpha }=\alpha /A_{I}$ if the reference set has an accuracy of $A_{I}$. This effect is consistent in both I and O. As for the set I, the real accuracy is $A_{I}$ while the estimated accuracy is $A_{I}/A_{I}$ = 100%. So, the estimation is inversely proportional to the accuracy of the reference set. This dependence on $A_{I}$ is very helpful to keep in mind when one interprets an actual assessment.

**Figure 3:**
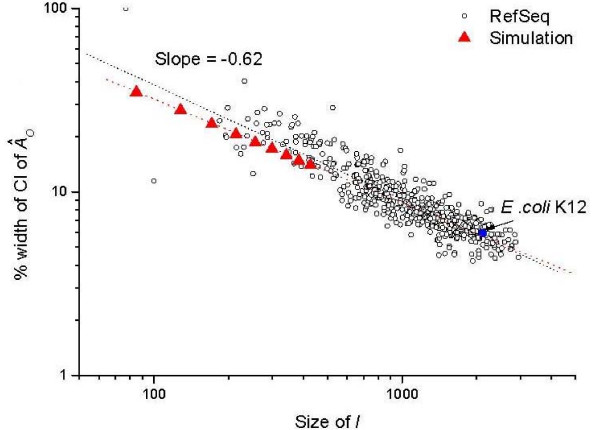
**Width of 95% CI of ${\hat{A}}_{O}$ as a function of the size of the reference set**. The 95% CIs of ${\hat{A}}_{O}$ are calculated for the testing set (triangles) and for the RefSeq annotation assessment (circles for 532 genomes and blue full circle for *E. coli *K12). The CIs are derived from 200 bootstrapping calculations in each case.

The above designed tests provide a unique opportunity to test if a bootstrapping strategy offers any knowledge about the uncertainty of the estimate. We carried out a bootstrapping calculation for the runs with $A_{O}$ = 60% and $A_{I}$ = 100%, the widths of the obtained 95% CIs are shown in Figure 3 as a function of $\Omega_{I}$ (by taking only a subset of genes from the set I). This dependence has an advantage to be compared to real assessment calculation. As shown, the width of the 95% CI follows approximately a power law dependence on $\Omega_{I}$, which is a result of the nonlinear optimization. At the largest set size of I, the width is around 13%, which is about 30% wider than that derived from the actual distribution of $\hat{\alpha }$ (which is around 10.0% for an approximate normal distribution with standard deviation 2.6%, as above). In other words, the bootstrapping calculation over-estimates the scattering of estimated $\alpha_{T}$, and hence it provides a good and conservative measure of the reliability of $\hat{\alpha }$. When we extended the 95% CI results for the testing to those in real assessment with the actual $\Omega_{I}$ in *E. coli*, we find that they agree remarkably well. This confirms the validity of the bootstrapping calculation. Generally speaking, Figure 3 shows that, for typical genomes with $\Omega_{I}$ ~1000 – 2000, the obtained assessment accuracy would have a width of 95% CI of 5% to 8% (equivalently ± 1% to ± 2% in standard deviation). This is rather a satisfactory outcome.

### Applications

#### Assessing RefSeq annotation

RefSeq is the most widely used public database on TIS, and its accuracy is the most concerned matter of this study. We have conducted an overall assessment on the TIS annotation for RefSeq. A total of 532 genomes are assessed. The annotation accuracy varies widely from 3.3% in *A. baumannii *ATCC 17978 to 96.8% in *P. pentosaceus *ATCC 25745 with an average of 80.6 ± 9.9%. About 40% of the genomes have accuracies higher than 85.0%, including genomes from several well studied genera such as *Bacillus*, *Escherichia*, *Salmonella *and *Pseudomonas*. In contrast, 13.5% of the genomes, most of which are GC-rich, have very suspicious TIS annotations with accuracies lower than 70%. A complete list of estimated accuracies for the 532 genomes is available in Additional File 2.

Below, we examined two annotation preferences that potentially contribute to the RefSeq annotation quality, namely tendencies to over-annotate LORF and to under-annotate ATG start codon.

**Figure 4:**
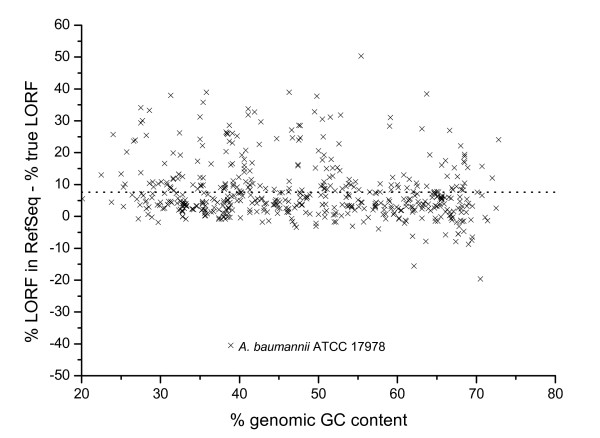
**Estimate of the excess of LORF from RefSeq**. The difference between annotated LORF and the true LORF estimated in our work shows the degree of over-annotation of LORF in RefSeq. The dot line shows the average.

As reported previously [7,8], RefSeq tends to over-annotate LORF. If the TIS annotation takes the rule of LORFs (*i.e*., always taking the 5'-most start codon), then its TIS accuracy would equal to the percentage of LORF in all true TISs (which will be referred below to as the percentage of true LORF). Our method can define a way to estimate this percentage of true LORF. For a genome for which we can generate a reliable reference set, then we can generate an artificial annotation by adopting the LORF rule. The final estimated accuracy of this artificial annotation is the percentage of true LORF. This method is applied to *Y. pestis*, and the estimated percentage of true LORF is 63.7%. The calculation of the actual percentage of LORF in the RefSeq annotation for *Y. pestis *is 92.6%. We then judge that there is about 30% over-annotation of LORF in this genome. This study is carried out for a total of 532 genomes, and the results are shown in Figure 4 where we found an average of 7.6 ± 9.1% over-annotated of LORFs in RefSeq.

**Figure 5:**
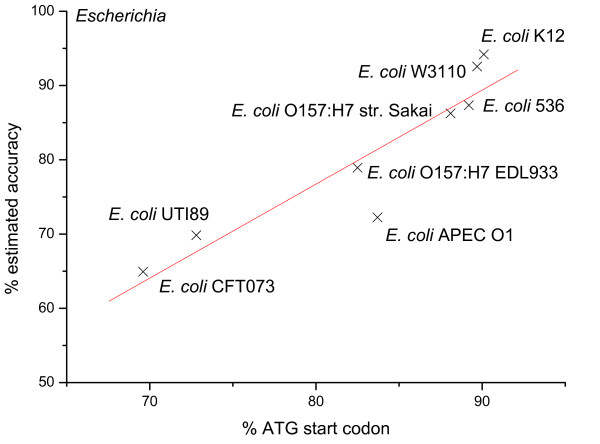
Correlation between RefSeq annotation accuracy and its ATG start codon usage for *E. coli*.

Another preference is the under-annotation of ATG start codon, for which we have now developed some statistical measures to provide further quantitative evidence. We have conducted calculation within genus, a taxonomic category ranking below family but above species. It is reasonable to expect that the TISs of species from the same genus show little difference in statistic such as the start codon usage. A total of 29 genera containing at least five selected genomes are studied, and the *Escherichia *genus is chosen to present our results; reported observations hold on most of the other genera (see Additional File 3). As shown in Figure 5, the percentage of annotated ATG start in the CFT073 strain is about 70%, whereas this percentage in the well-studied K12 strain reaches a much higher value of 90%. Note that the percentage calculated from the confirmed *EcoGen854 *data set is about 91%. Our estimated accuracy of TIS annotation for CFT073 strain is below 70%, significantly lower than the K12 strain (about 94%). Figure 5 shows a clear linear correlation between the ATG start codon usage and the accuracy for all strains in the *Escherichia *genus.

#### Assessing other TIS annotations

**Figure 6:**
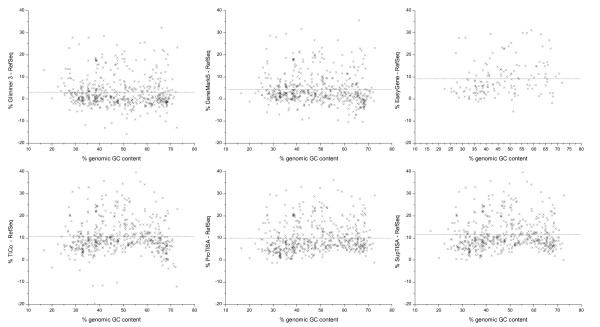
**Annotation accuracy comparison between RefSeq and six other annotations**. The dot line shows the average.

Since our reference set is constructed with the intersection of all relevant TIS databases/predictors, it is not biased towards any one, and hence we can carry out the analysis of accuracy for all of the predictors for the 532 genomes. This subsection is devoted to a discussion of their performances. We chose RefSeq as a standard of accuracy comparison for presenting the results. To reduce false positives, genes not annotated by RefSeq and genes with length short than 300 bps were excluded, as implied in [8,18]. Figure 6 shows the accuracy difference of the five other TIS predictors to RefSeq predictions.

As two of the most popular gene-finders, Glimmer and GeneMark have been used to annotate hundreds of genomes. The most recent versions, Glimmer 3 and GeneMarkS, include a RBS model to predict TISs, which is in a form of PWM whose parameters are derived by a Gibbs sampler. High performances are reported on two well-studied genomes *E. coli *K12 and *B. subtilis *[7,16]. When assessed here on the 532 genomes by our method, Glimmer 3 and GeneMarkS report average accuracies of 83.6 ± 7.4% and 85.0 ± 7.3%, respectively, which are higher than RefSeq (80.6 ± 9.9%). However, for a considerable numbers of genomes, Glimmer 3 and GeneMarkS have made limited or no improvement over RefSeq (see Figure 6).

EasyGene has only published 157 genomes [21] and is believed to be a better TIS resource than RefSeq [8]. Indeed, our assessment confirmes that, for its reported set of genomes, EasyGene's annotation has a noticeably high average accuracy of 86.7 ± 6.3%, which is about 10% higher than the RefSeq prediction for the same set (see Figure 6). Note that EasyGene is reported to make conservative choices in gene prediction [8,15], and it excludes often 5–10% genes of RefSeq, some of which have function annotation.

Unlike gene finders, TiCo is a post-processor of an existent annotation. High performance was reported on *E. coli *K12 and *B. subtilis*, as well as on GC rich genomes such as *P. aeruginosa *PAO1 [17]. As shown in Figure 6, the improvement on RefSeq is indeed remarkable, with an average improvement on accuracy of 10% over all 532 genomes. Note, however, that the accuracy improvement is relatively lower in GC-rich and AT-rich genomes (in the wing part of Figure 6).

ProTISA is a recently published database dedicated to TIS annotation in prokaryotic genomes. It is generated by collecting various confirmed TISs and predictions from MED-Start (upgraded), which post-processes the RefSeq annotation [14,22,23]. The assessment carried out here indicates that, over 532 genomes, the ProTISA has a mean accuracy of 90.5%, which is 9.9% higher than RefSeq (see Figure 6). As a more rigorous comparison, we applied the paired-samples t-test to judge if ProTISA gives a significantly higher accuracy than RefSeq, and obtained a t-value of 31.2, much larger than 1.7 at 95% confidence and for degrees of 531. Thus, a definite positive answer is derived. Besides, there are 101 genomes for which ProTISA's prediction accuracy is higher by 15% than RefSeq; for these genomes, the RefSeq shows a clear preference of over-annotating LORF and under-annotating ATG start codon (data not shown). In addition to accurate TIS annotation, ProTISA annotates potential regulatory signals, which are helpful in investigating the diversity of translation initiation mechanism. For example, besides SD signal, Pribnow box is found at 10 bps upstream to TIS in many bacteria genomes, suggesting that leaderless gene may not be rare in bacteria [14].

#### A new TIS database: SupTISA

The method of evaluation proposed in this paper is based on a fundamentally different principle, the principle of homogeneity for the PWMs of any subset of genome as a linear combination of three elementary PWMs. This principle is based on the universal process of gene translation, and it is a macroscopic property for the ensemble of TISs. This information is supplementary to the properties that are used by TIS predictors, and hence can (and should) be used to provides a complementary way for achieving the global annotation performance. In other words, we propose to construct a new TIS annotation database by selecting the best TIS predictor's annotation for any given genome; the resulting annotations organize a new database (of 532 genomes at present) and is named SupTISA [24]. This is possible because the assessment is totally independent and unbiased.

Specifically, for each genome, SupTISA selects the one of RefSeq, ProTISA, EasyGene, GeneMarkS, Glimmer 3 and TiCo with the highest accuracy as the SupTISA annotation and provides its downloading at the web address [24] for TIS annotations. SupTISA takes advantage of the complementary quality of all the existing TIS predictors. For instance, ProTISA are generally better than TiCo on genomes with biased GC content, but TiCo outperforms ProTISA on others (data not shown). The result is that SupTISA achieves an average annotation accuracy of 92.1 ± 4.7%. Figure 6 shows that SupTISA generally over-performs RefSeq by 5% to 30%. Therefore, SupTISA provides the best resource for experimental use and for computational study related to TIS.

## Discussions and Conclusion

Translation is a fundamental process for an organism, and the regulatory signals relevant to this process should have relatively uniform distribution across a genome. A PWM of aligned sequences around TIS summarizes the statistical information of the signal, and is then a tool to use for study how much, in a given set of annotation, the true signal has contributed. This is the principle we use for inventing, for the first time, an algorithm for large-scale evaluation of TIS's prediction accuracy. The work done on the testing with confirmed genes and on assessing six databases/predictors over 532 genomes give rise to a series of consistent results. Although the actual accuracy results may be subject to a few percents of uncertainty, due to statistical fluctuations of finite sample sizes and possible distortion of the reference sets, the assessments seem to be a valid leading order measure of the TIS annotations. Such assessment is meaningful, especially when the estimated accuracy is low: typically, some unjustified or simplified assumptions are used during the annotation. Our assessment then provides a tool for experimental or computational biologists to avoid to be mis-led by an over-simplified annotation. We have shown that the RefSeq annotations for some genomes are of this nature.

Correct annotation is important to both *in vivo *and *in silico *studies of translation initiation. In *P. horikoshii *OT3 and several other archaeal genomes, Cang and Wang [25] reported a high frequency of ATG triplets at 9 bps downstream of annotated TISs using GenBank's TIS annotation data. It was suggested that "a remedial initiation site for archaea ... reflect the decreased effciency of the translation initiation machinery in archaea". However, after taking a refined dataset of TIS annotations from the present database, such unexpected over-frequency disappeared (data not shown). It is then likely that the observed excess of ATG triplets is due to the excess of false TIS upstream to the true TISs present in the study. As an additional outcome, the present assessment yields a new database, called SupTISA. The interest of SupTISA lies in the fact that none of the TIS predictors is able to correctly take into account all properties of sequences around TIS over the entire family of prokaryotic genomes, and hence SupTISA can integrate them as a more macro-selector. This is achieved because SupTISA is based on a macroscopic principle (over all genomes) of homogeneity of translation machinery.

## Authors' contributions

ZSS and GQH conceived the study, designed the applications and drafted the manuscript, ZSS and HQZ co-supervised the development of the work, XBZ and GQH designed and implemented the algorithm. LNJ performed part of the test. All authors read and approved the final manuscript.

## Supplementary Material

Additional file 1 **Supplementary details of the method**. Details for deducing Eq. 6 and minimizing the sum of squared errors in Eq. 7. Click here for file

Additional file 2 **Estimated TIS annotation accuracies of six selected databases/predictors**. Accuracies of TIS annotation on a total of 532 genomes for RefSeq, Glimmer 3, GeneMarkS, EasyGene, TiCo and ProTISA. Click here for file

Additional file 3 **Correlation between annotation accuracy and ATG start codon usage**. A total of 29 genera were selected. The linear fit was applied if the Pearson Correlation is significant at 95% confidence. Click here for file

## References

[B1] Shine J, Dalgarno L (1974). The 3'-terminal sequence of *E. coli *16S RNA: complementarity to nonsense triplets and ribosome binding sites. Proc Natl Acad Sci U S A.

[B2] Londei P (2005). Evolution of translational initiation: new insights from the archaea. FEMS Microbiol Rev.

[B3] Torarinsson E, Klenk HP, Garrett RA (2005). Divergent transcriptional and translational signals in Archaea. Environ Microbiol.

[B4] Gold L (1988). Posttranscriptional regulatory mechanisms in *Escherichia Coli*. Annu Rev Biochem.

[B5] Zhu HQ, Hu GQ, Yang YF, Wang J, She ZS (2007). MED: a new non-supervised gene prediction algorithm for bacterial and archaeal genomes. BMC Bioinformatics.

[B6] Poole FL, Gerwe BA, Hopkins RC, Schut GJ, Weinberg MV, Jenney FEJ, Adams MW (2005). Defining genes in the genome of the hyperthermophilic Archaeon *Pyrococcus furiosus*: implications for all microbial genomes. J Bacteriol.

[B7] Besemer J, Lomsadze A, Borodovsky M (2001). GeneMarkS: a self-training method for prediction of gene starts in microbial genomes. Implications for finding sequence motifs in regulatory regions. Nucleic Acids Res.

[B8] Nielsen P, Krogh A (2005). Large-scale prokaryotic gene prediction and comparison to genome annotation. Bioinformatics.

[B9] Starmer J, Stomp A, Vouk M, Bitzer D (2006). Predicting Shine-Dalgarno sequence locations exposes genome annotation errors. PLoS Comput Biol.

[B10] Frishman D, Mironov A, Gelfand M (1999). Starts of bacterial genes: estimating the reliability of computer predictions. Gene.

[B11] Gorodkin J, Heyer LJ, Brunak S, Stormo GD (1997). Displaying the information contents of structural RNA alignments: the structure logos. Comput Appl Biosci.

[B12] Rudd KE (2000). EcoGene: a genome sequence database for *Escherichia coli *K-12. Nucleic Acids Res.

[B13] Pruitt K, Tatusova T, Maglott D (2007). NCBI reference sequences (RefSeq): a curated non-redundant sequence database of genomes, transcripts and proteins. Nucleic Acids Res.

[B14] Hu GQ, Zheng XB, Yang YF, Ortet P, She ZS, Zhu HQ (2008). ProTISA: a comprehensive resource for translation initiation site annotation in prokaryotic genome. Nucleic Acids Res.

[B15] Larsen TS, Krogh A (2003). EasyGene – a prokaryotic gene finder that ranks ORFs by statistical significance. BMC Bioinformatics.

[B16] Delcher AL, Bratke KA, Powers EC, Salzberg SL (2007). Identifying bacterial genes and endosymbiont DNA with Glimmer. Bioinformatics.

[B17] Tech M, Meinicke P (2006). An unsupervised classification scheme for improving predictions of prokaryotic TIS. BMC Bioinformatics.

[B18] Skovgaard M, Jensen LJ, Brunak S, Ussery D, Krogh A (2001). On the total number of genes and their length distribution in complete microbial genomes. Trends Genet.

[B19] Zhang CT, Zhang R (1991). Analysis of distribution of bases in the coding sequences by a diagrammatic technique. Nucleic Acids Res.

[B20] Staden R (1984). Computer methods to locate signals in nucleic acid sequences. Nucleic Acids Res.

[B21] EasyGene. http://servers.binf.ku.dk/cgi-bin/easygene/search.

[B22] Zhu HQ, Hu GQ, Ouyang ZQ, Wang J, She ZS (2004). Accuracy improvement for identifying translation initiation sites in microbial genomes. Bioinformatics.

[B23] ProTISA. http://mech.ctb.pku.edu.cn/protisa/.

[B24] SupTISA. http://mech.ctb.pku.edu.cn/protisa/SupTISA/.

[B25] Cang XH, Wang J (2004). A unique ATG triplet downstream of gene start in archaea: implications for translation initiation and evolution. Gene.

