# Supplementary material for: Computational evaluation of TIS annotation for prokaryotic genomes
Source: BMC Bioinformatics. 2008 Mar 25;9:160. doi: 10.1186/1471-2105-9-160 (PMC2362131; doi:10.1186/1471-2105-9-160)
Supplement: Additional file 1 — Supplementary details of the method. Details for deducing Eq. 6 and minimizing the sum of squared errors in Eq. 7. [file 1471-2105-9-160-S1.pdf]

Contents

1 Calculating the variance of ε 1

2 Minimizing the weighted sum of squared errors $\varepsilon'^T\Sigma'^-\varepsilon'$ 3

1 Calculating the variance of ε

The set $\mathbb{O}$ is a mixture of three types of TISs $\mathbb{T}$, $\mathbb{F}_u$ and $\mathbb{F}_d$, we have

$$\hat{W}_{\mathbb{O}}^{(\mathbb{O})} = \alpha_{\mathbb{T}}\hat{W}_{\mathbb{T}}^{(\mathbb{O})} + \alpha_{\mathbb{F}_u}\hat{W}_{\mathbb{F}_u}^{(\mathbb{O})} + \alpha_{\mathbb{F}_d}\hat{W}_{\mathbb{F}_d}^{(\mathbb{O})}, \tag{1}$$

where the superscript $(\mathbb{O})$ refers to PWMs obtained from the set $\mathbb{O}$ and three PWMs $\hat{W}_{\mathbb{T}}^{(\mathbb{O})}$, $\hat{W}_{\mathbb{F}_u}^{(\mathbb{O})}$ and $\hat{W}_{\mathbb{F}_d}^{(\mathbb{O})}$ are virtually calculated from the three types of TISs in the set $\mathbb{O}$. Since we don't know these three PWMs, we use three other PWMs obtained from the set $\mathbb{I}$ to replace them, and an error ε is generated

$$\hat{W}_{\mathbb{O}}^{(\mathbb{O})} = \alpha_{\mathbb{T}}\hat{W}_{\mathbb{T}}^{(\mathbb{I})} + \alpha_{\mathbb{F}_u}\hat{W}_{\mathbb{F}_u}^{(\mathbb{I})} + \alpha_{\mathbb{F}_d}\hat{W}_{\mathbb{F}_d}^{(\mathbb{I})} + \varepsilon, \tag{2}$$

Here we arrange the $4 \times (l + r)$ matrices in row order to $4(l + r)$-dimension vectors. Consequently, $W_j(\mu)$ (in main text) becomes $W(4(j - 1) + \mu)$, $j = 1, 2, ..., l + r$ and $\mu = 1, 2, 3, 4$.

Thus the error term ε can be explicitly written as

$$\varepsilon = \sum_{i=1}^{3} \alpha_i(\hat{W}_i^{(\mathbb{O})} - \hat{W}_i^{(\mathbb{I})}), \tag{3}$$

where the index i=1,2,3 refer to the three sets $\mathbb{T}$, $\mathbb{F}_u$ and $\mathbb{F}_d$, respectively. The "homogeneity assumption" (see the paper) says that $\hat{W}_i^{(\mathbb{O})}$ and $\hat{W}_i^{(\mathbb{I})}$ are independent finite-sample estimations of the same PWM W_i. Therefore,

$$E(\varepsilon) = \sum_i \alpha_i(E(\hat{W}_i^{(\mathbb{O})}) - E(\hat{W}_i^{(\mathbb{I})})) = \sum_i \alpha_i(W_i - W_i) = 0, \tag{4}$$

and the variance of ε can be written as

$$Var(\varepsilon) = \sum_i \alpha_i^2(Var(\hat{W}_i^{(\mathbb{O})}) + Var(\hat{W}_i^{(\mathbb{I})})). \tag{5}$$

We further assume that the nucleotide frequencies at different positions in the PWM are independent (Staden, R. (1984) Computer methods to locate signals in nucleic acid sequences. *Nucleic Acids Res*, **12** :505-519). Thus, for position j and position k (where j, k=1, 2, ..., $l + r$), we have

$$\begin{aligned} &Cov(\hat{W}_i^{(\mathbb{O})}(4(j-1)+\mu), \hat{W}_i^{(\mathbb{O})}(4(k-1)+\nu)) \\ &\quad = \frac{W_i(4(j-1)+\mu)\delta_{\mu,\nu} - W_i(4(j-1)+\mu)W_i(4(k-1)+\nu)}{\alpha_i\Omega_{\mathbb{O}}}\delta_{j,k} \end{aligned} \tag{6}$$

and

$$
\begin{aligned}
&Cov(\hat{W}_i^{(\mathbb{I})}(4(j-1)+\mu), \hat{W}_i^{(\mathbb{I})}(4(k-1)+\nu)) \\
&\quad = \frac{W_i(4(j-1)+\mu)\delta_{\mu,\nu} - W_i(4(j-1)+\mu)W_i(4(k-1)+\nu)}{\Omega_i}\delta_{j,k},
\end{aligned}
\tag{7}
$$

where μ, ν=1, 2, 3, 4, denoting nucleotide A, C, G, T, respectively. This yields

$$
Var(\hat{W}_i^{(\mathbb{O})}) = \frac{1}{\alpha_i \Omega_{\mathbb{O}}}\Sigma_i \tag{8}
$$

and

$$
Var(\hat{W}_i^{(\mathbb{I})}) = \frac{1}{\Omega_i}\Sigma_i, \tag{9}
$$

where Σ_i is a block diagonal symmetric matrix and the number of blocks is determined by the number of positions of the PWM alignment. An block according to position j is shown as below:

$$
\begin{pmatrix}
W_i(4j-3)-W_i^2(4j-3) & -W_i(4j-3)W_i(4j-2) & -W_i(4j-3)W_i(4j-1) & -W_i(4j-3)W_i(4j) \\
\cdot & W_i(4j-2)-W_i^2(4j-2) & -W_i(4j-2)W_i(4j-1) & -W_i(4j-2)W_i(4j) \\
\cdot & \cdot & W_i(4j-1)-W_i^2(4j-1) & -W_i(4j-1)W_i(4j) \\
\cdot & \cdot & \cdot & W_i(4j)-W_i^2(4j)
\end{pmatrix}.
$$

With Eq. 5, Eq. 8 and Eq. 9, we finally obtain the variance of ε

$$
Var(\varepsilon) = \sum_i (\frac{\alpha_i^2}{\Omega_i} + \frac{\alpha_i}{\Omega_{\mathbb{O}}})\Sigma_i \tag{10}
$$

Then we reduce data redundancy in the PWM to make $Var(\varepsilon)$ full rank with a Z-transformation as below (Zhang, C.T. and Zhang, R. (1991) Analysis of distribution of bases in the coding sequences by a diagrammatic technique. *Nucleic Acids Res*, **19** :6313-6317)

$$
\begin{cases}
V(3j-2) = W(4j-3) + W(4j-2) - W(4j-1) - W(4j) \\
V(3j-1) = W(4j-3) - W(4j-2) + W(4j-1) - W(4j) \\
V(3j) = W(4j-3) - W(4j-2) - W(4j-1) + W(4j).
\end{cases}
$$

Consequently,

$$
\hat{V}_{\mathbb{O}} = \sum_i \alpha_i \hat{V}_i + \varepsilon'. \tag{11}
$$

The variance of ε' has a similar form with ε

$$
Var(\varepsilon') = \sum_i (\frac{\alpha_i^2}{\Omega_i} + \frac{\alpha_i}{\Omega_{\mathbb{O}}})\Sigma_i', \tag{12}
$$

where $\Sigma_i' = H\Sigma_i H^T$ and H is a block diagonal matrix with each block being

$$\begin{pmatrix} 1 & 1 & -1 & -1 \\ 1 & -1 & 1 & -1 \\ 1 & -1 & -1 & 1 \end{pmatrix}.$$

$\hat{W}_i$ is used as an estimate of W_i to calculate Σ_i'. The effect of this approximation is of high order in our model if the samples are sufficient, for example over 50. In the following part, $Var(\varepsilon')$ will be denoted by Σ' for convenience.

2 Minimizing the weighted sum of squared errors $\varepsilon'^T\Sigma'^-\varepsilon'$

In the main text we mentioned that α is estimated by minimizing the weighted sum of square errors

$$f(\alpha_1,\alpha_2,\alpha_3) = (\hat{V}_{\mathbb{O}} - \sum_{i=1}^{3}\alpha_i\hat{V}_i)^T\Sigma'^-(\hat{V}_{\mathbb{O}} - \sum_{i=1}^{3}\alpha_i\hat{V}_i) \tag{13}$$

, where the index i=1,2,3 refer to the three sets $\mathbb{T}$, $\mathbb{F}_u$ and $\mathbb{F}_d$ respectively.

Substitute α_3 with

$$\alpha_3 = 1 - \sum_{i=1}^{2}\alpha_i \tag{14}$$

and Eq. 13 can be written as

$$f(\alpha_1,\alpha_2) = (S - \sum_{i=1}^{2}\alpha_i T_i)^T\Sigma'^-(S - \sum_{i=1}^{2}\alpha_i T_i) \tag{15}$$

where

$$S = \hat{V}_{\mathbb{O}} - \hat{V}_3 \tag{16}$$

and

$$T_i = \hat{V}_i - \hat{V}_3,\ i = 1,2 \tag{17}$$

To minimize $f(\alpha_1,\alpha_2)$, we let the partial derivatives be zero

$$\frac{\partial f}{\partial \alpha_j} = -2T_j^T\Sigma'^-(S - \sum_{i=1}^{2}\alpha_i T_i) - (S - \sum_{i=1}^{2}\alpha_i T_i)^T\Sigma'^-\frac{\partial \Sigma}{\partial \alpha_j}\Sigma'^-(S - \sum_{i=1}^{2}\alpha_i T_i) = 0 \tag{18}$$

Eq. 18 can be simplified to

$$\sum_{i=1}^{2}K_{ij}\alpha_i = L_j, j = 1,2 \tag{19}$$

where

$$K_{ij} = T_j^T\Sigma'^-T_i,\ i,j = 1,2 \tag{20}$$

and

$$L_j = T_j^T \Sigma'^{-} S + \frac{1}{2}(S - \sum_{i=1}^{2} \alpha_i T_i)^T \Sigma'^{-} \frac{\partial \Sigma'}{\partial \alpha_j} \Sigma'^{-} (S - \sum_{i=1}^{2} \alpha_i T_i).\ j = 1, 2 \tag{21}$$

There are 2 equations and 2 variables. The equations can be solved iteratively. First we set

$$\alpha_i^{(0)} = 1/3, i = 1, 2 \tag{22}$$

Then we calculate $\Sigma'^{(0)}$ and $\partial \Sigma'^{(0)} / \partial \alpha_j$ by Eq. 12 ,$K_{ij}^{(0)}$and$L_j^{(0)}$ by Eq. 20 and Eq. 21, and then obtain $\alpha^{(1)}$ by solving

$$\sum_{i=1}^{2} K_{ij}^{(0)} \alpha_i^{(1)} = L_j^{(0)},\ j = 1, 2 \tag{23}$$

Then $\alpha_i^{(1)}$ is used to calculate $\alpha_i^{(2)}$ and the process is repeated until $\sum_{i=1}^{2} |\alpha_i^{(n)} - \alpha_i^{(n-1)}| < 10^{-6}$.

It's difficult to prove that this iteration process will always converge, but in practice it converges quite fast. For instance, when to estimate the accuracy of RefSeq annotation for *E. coli* K12, the algorithm converge in less than 10 steps (Fig. 1); we also show the iteration process of another 12 randomly selected genomes in Fig. 2.

[Fig. 1 about here.]

[Fig. 2 about here.]

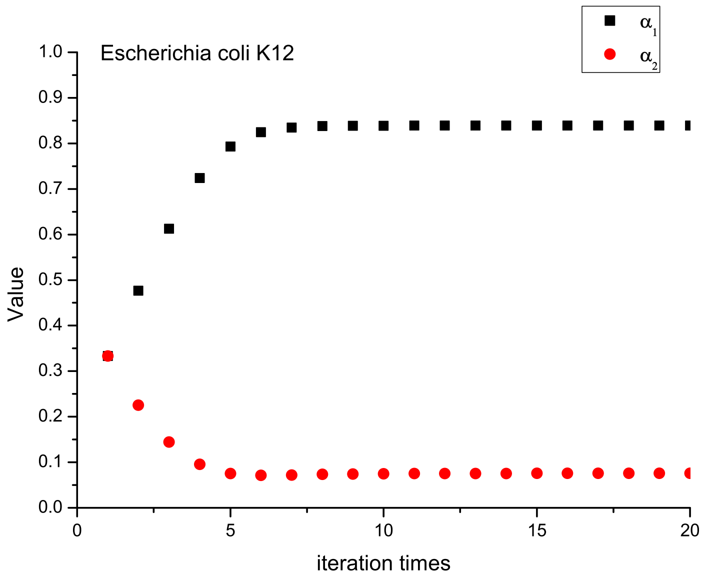

Fig. 1. The convergency of the optimization algorithm (shown on *E. coli* K12).

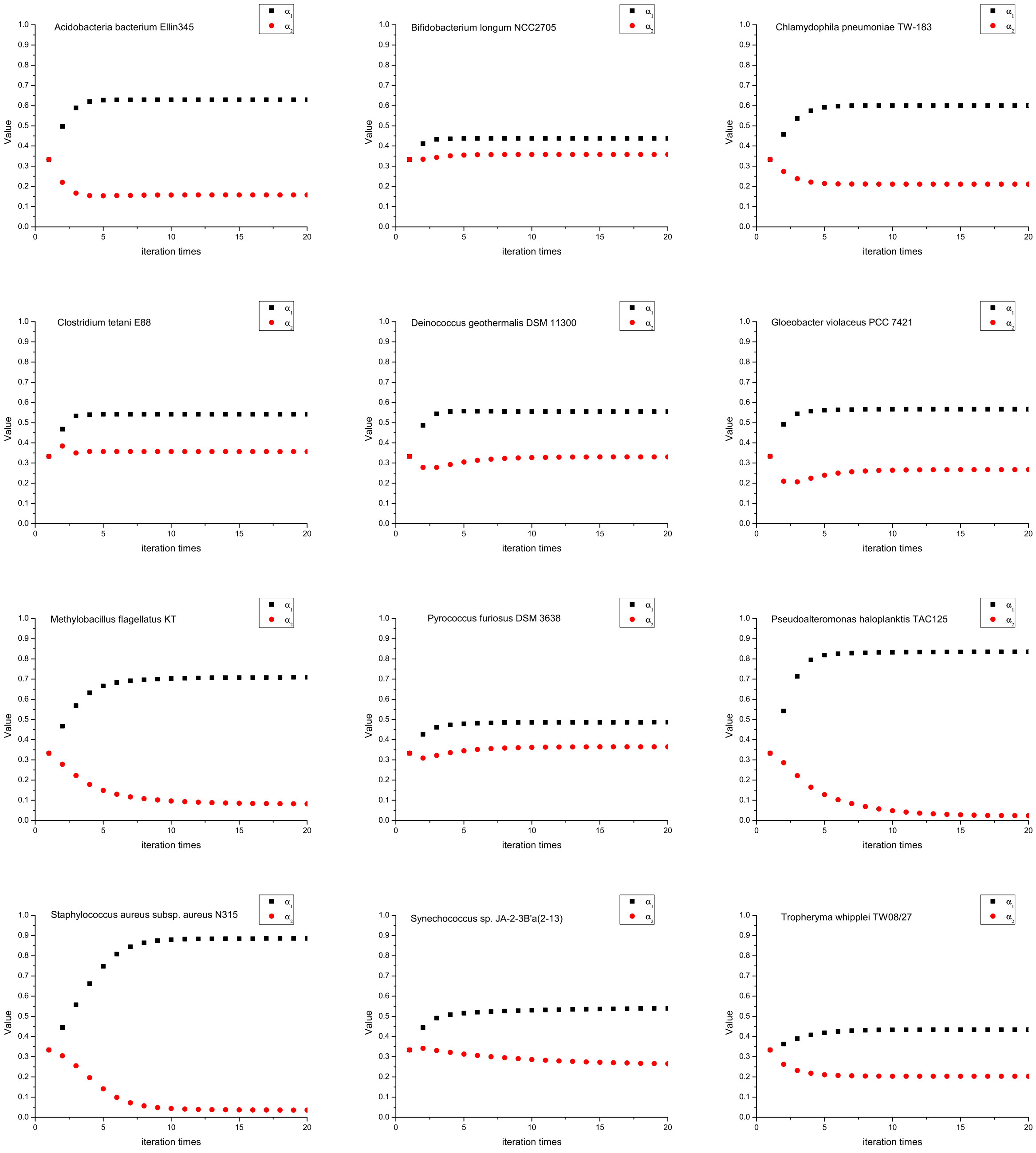

Fig. 2. The convergency of the optimization algorithm (shown on 12 randomly selected genomes).
