# Supplementary figures and images for: Computational evaluation of TIS annotation for prokaryotic genomes
Source: BMC Bioinformatics. 2008 Mar 25;9:160. doi: 10.1186/1471-2105-9-160 (PMC2362131; doi:10.1186/1471-2105-9-160)

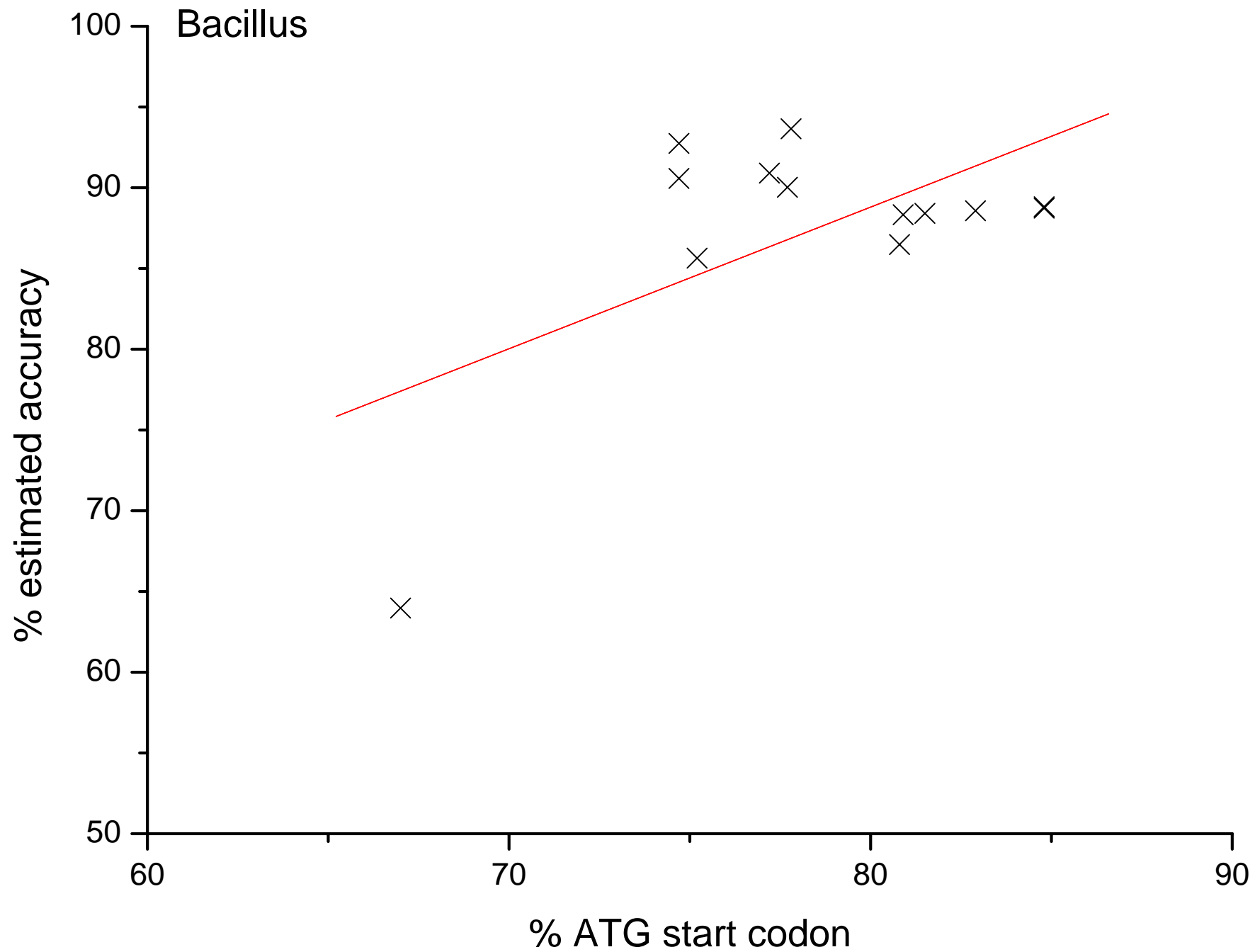

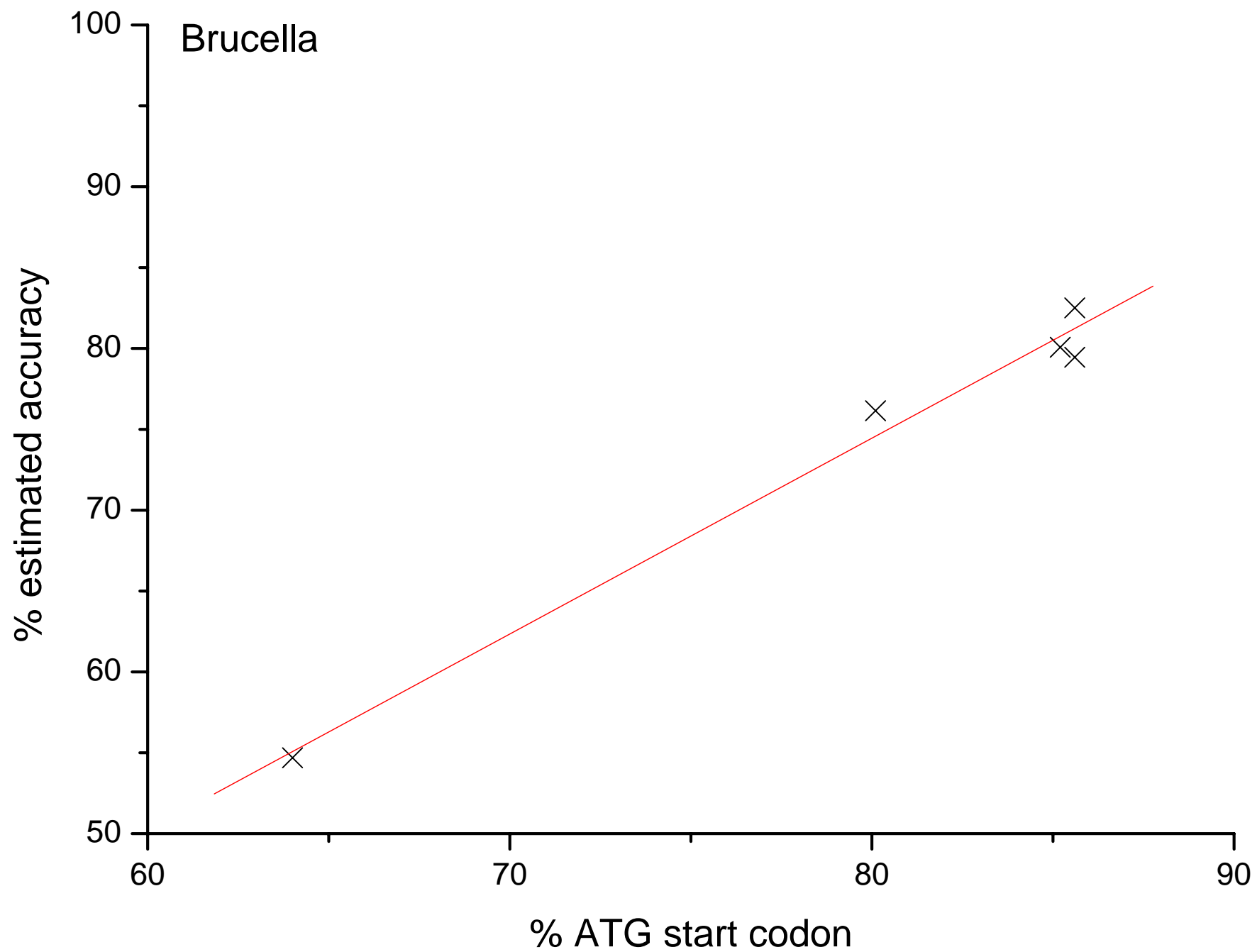

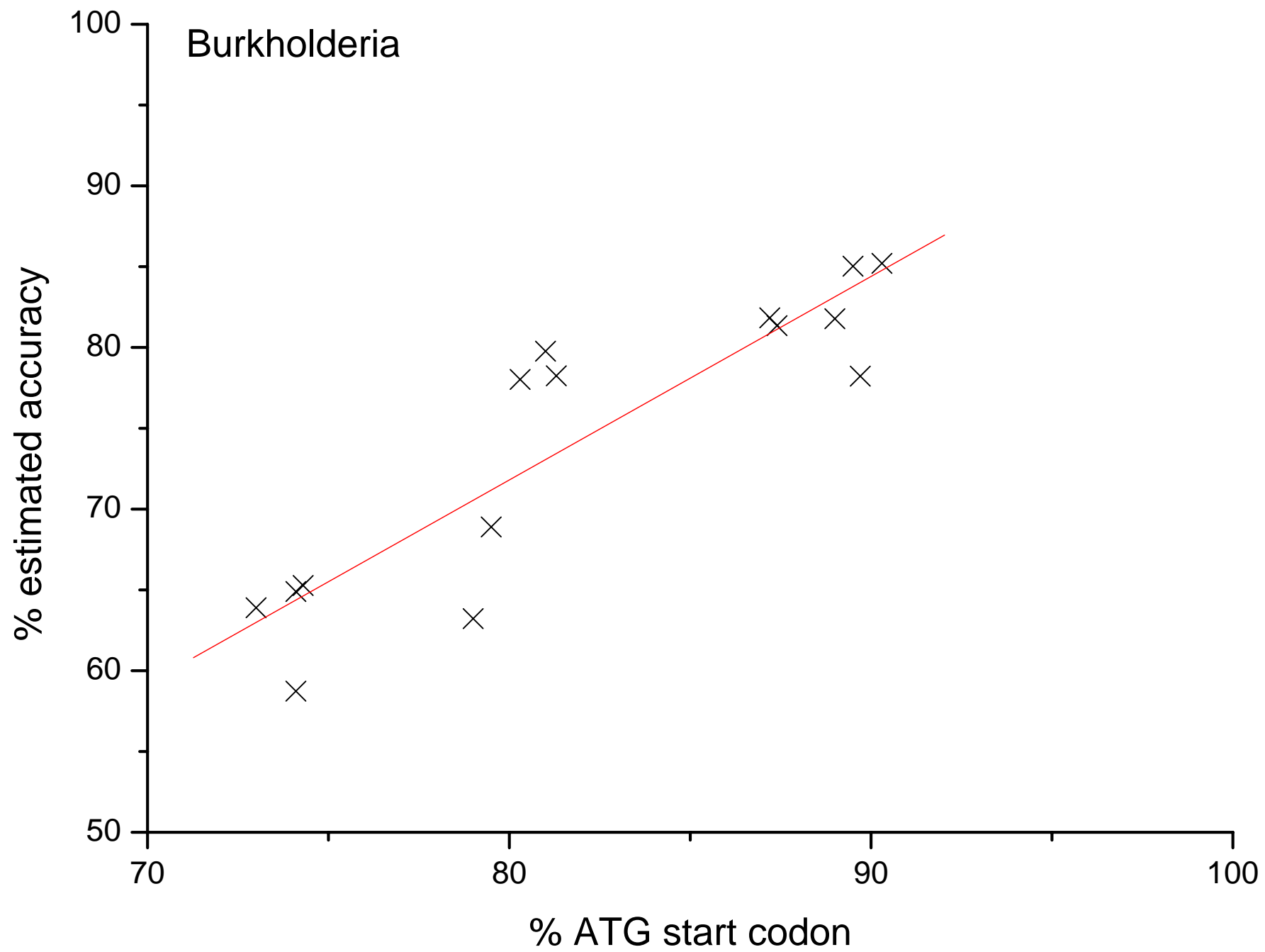

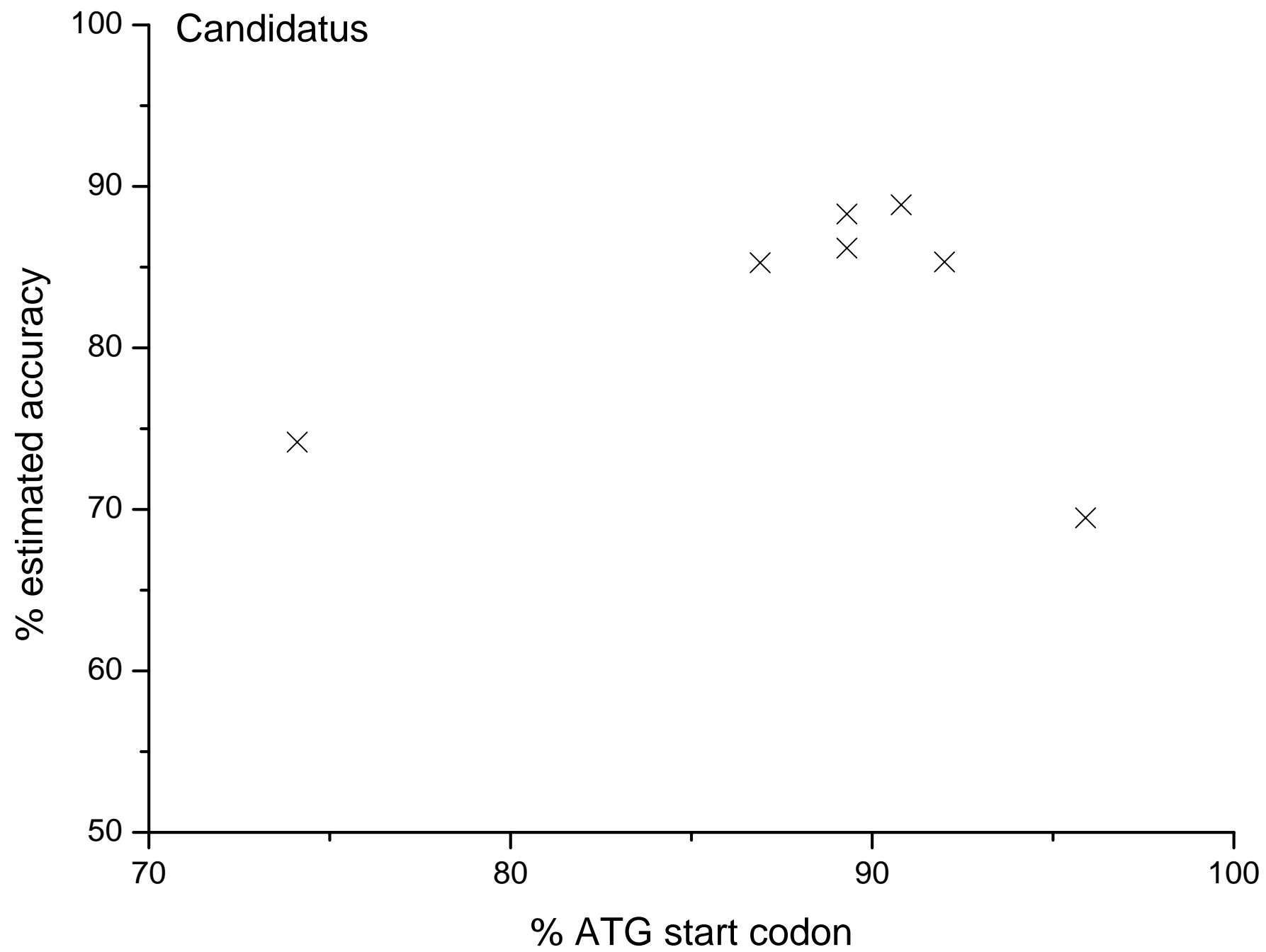

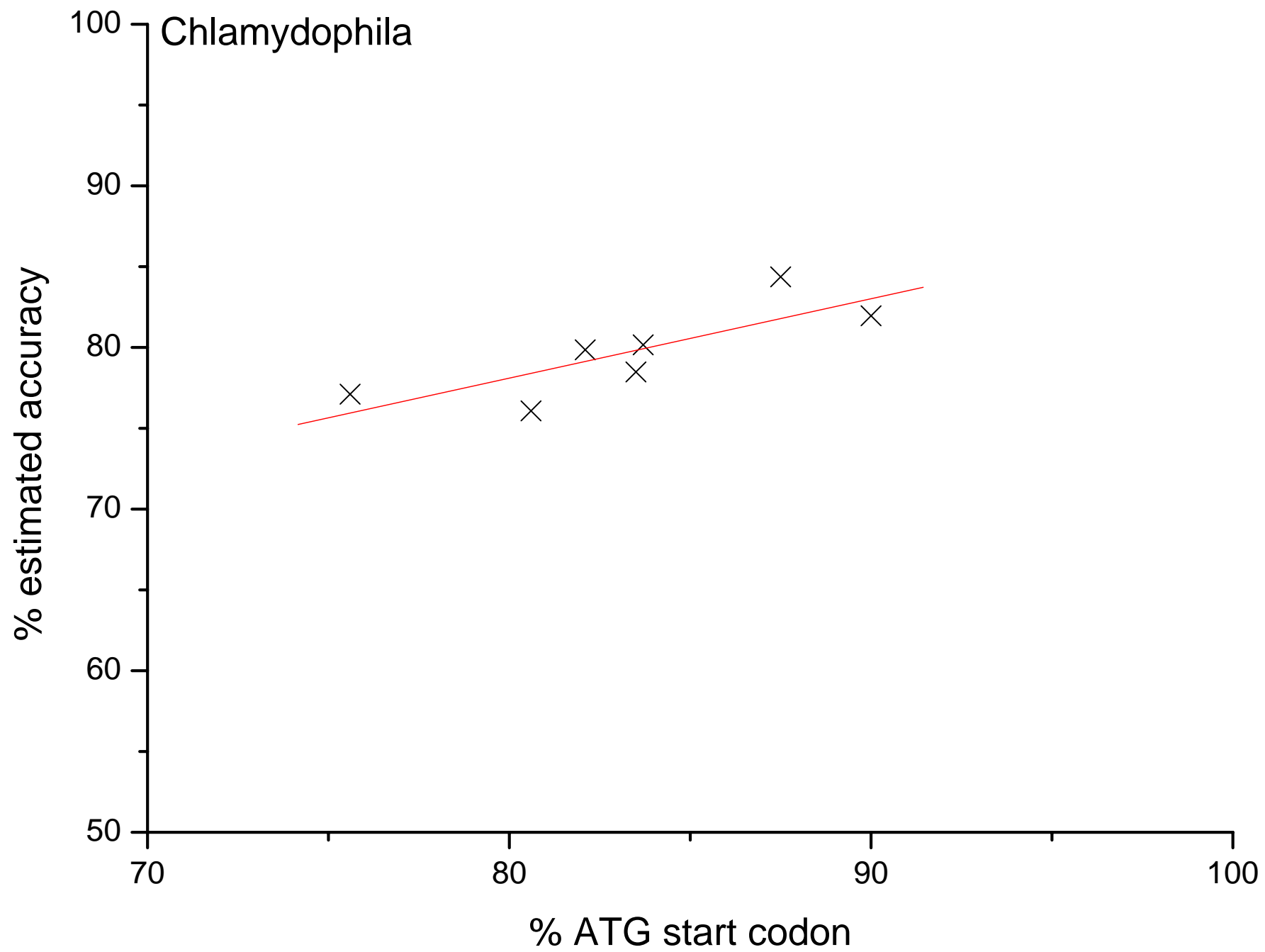

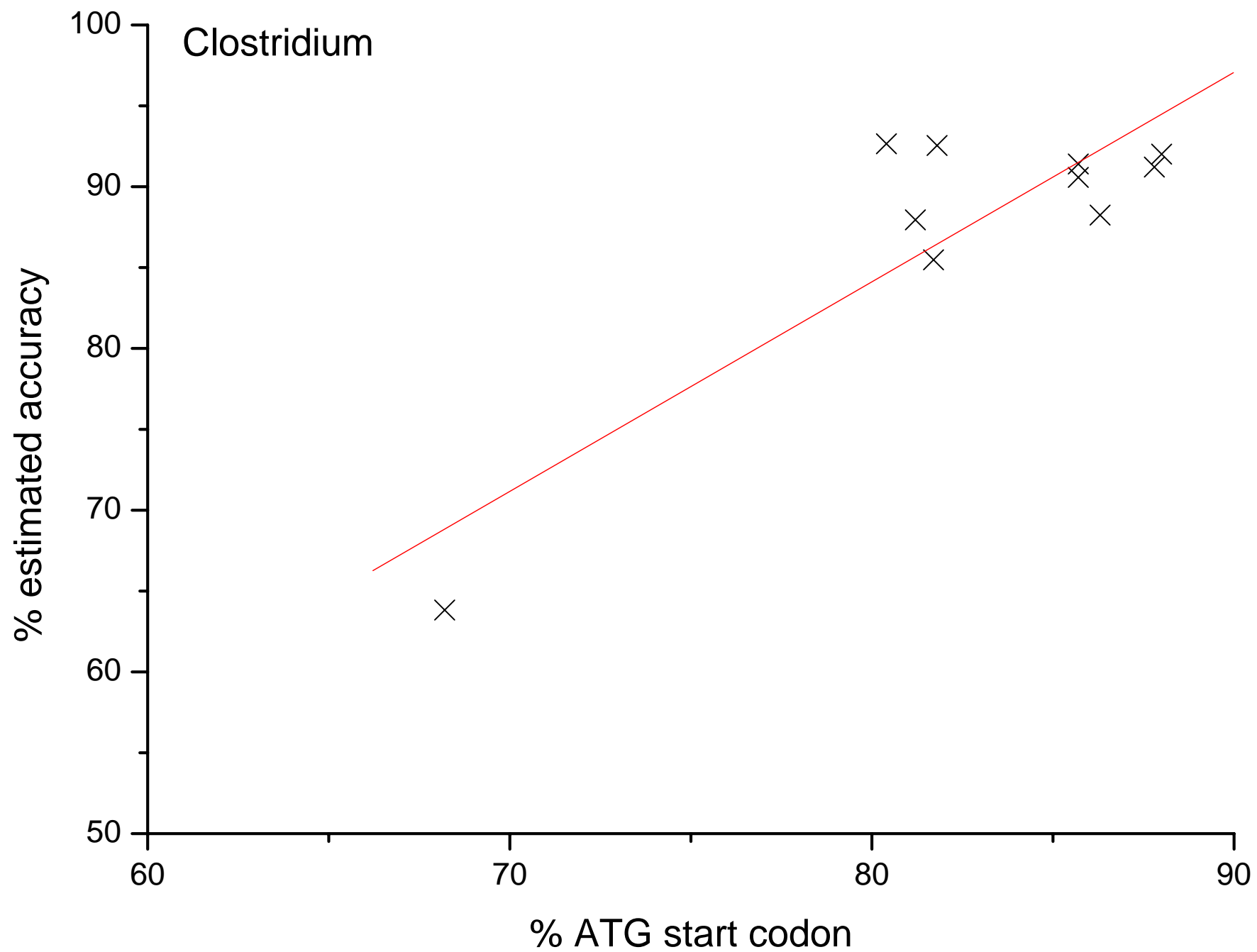

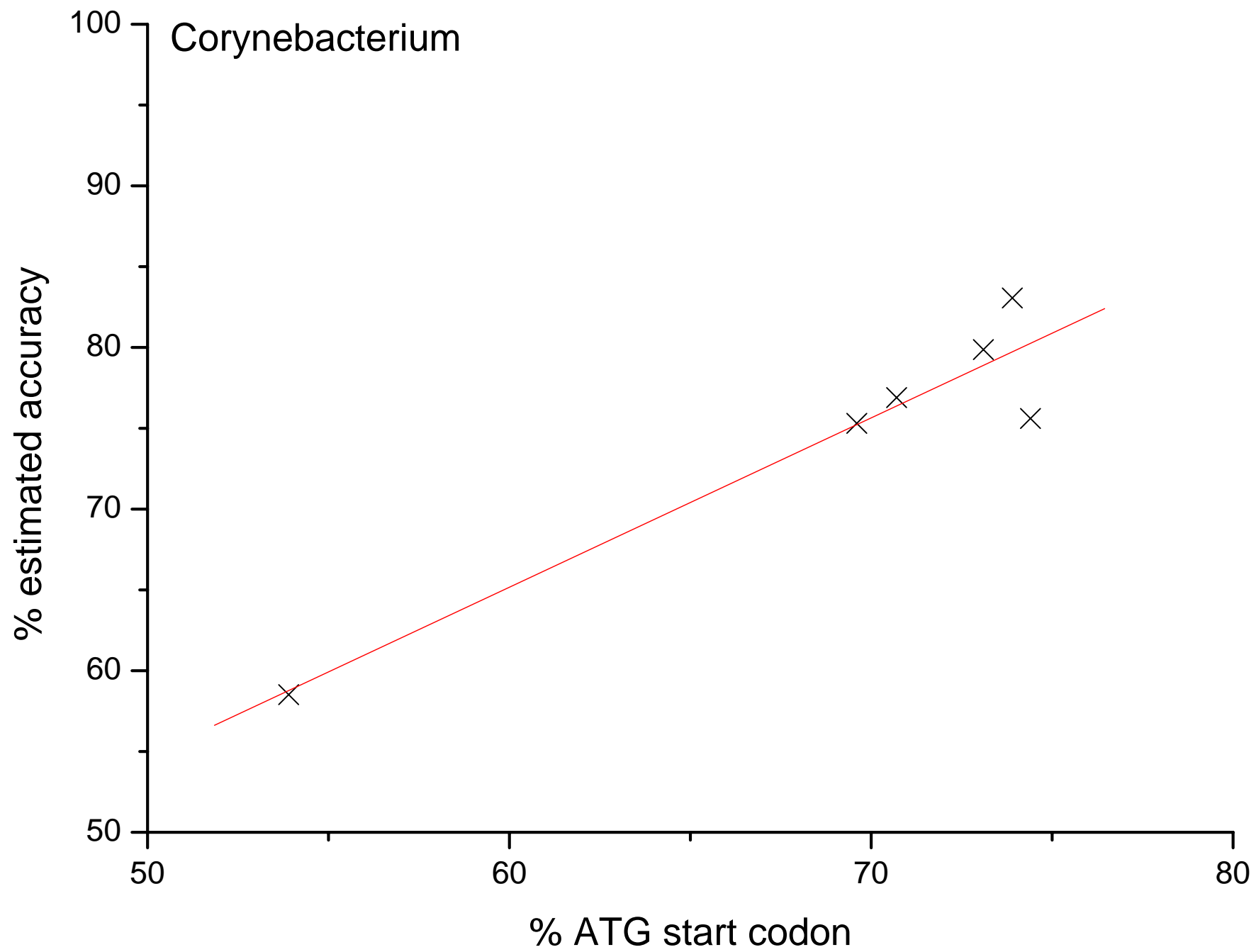

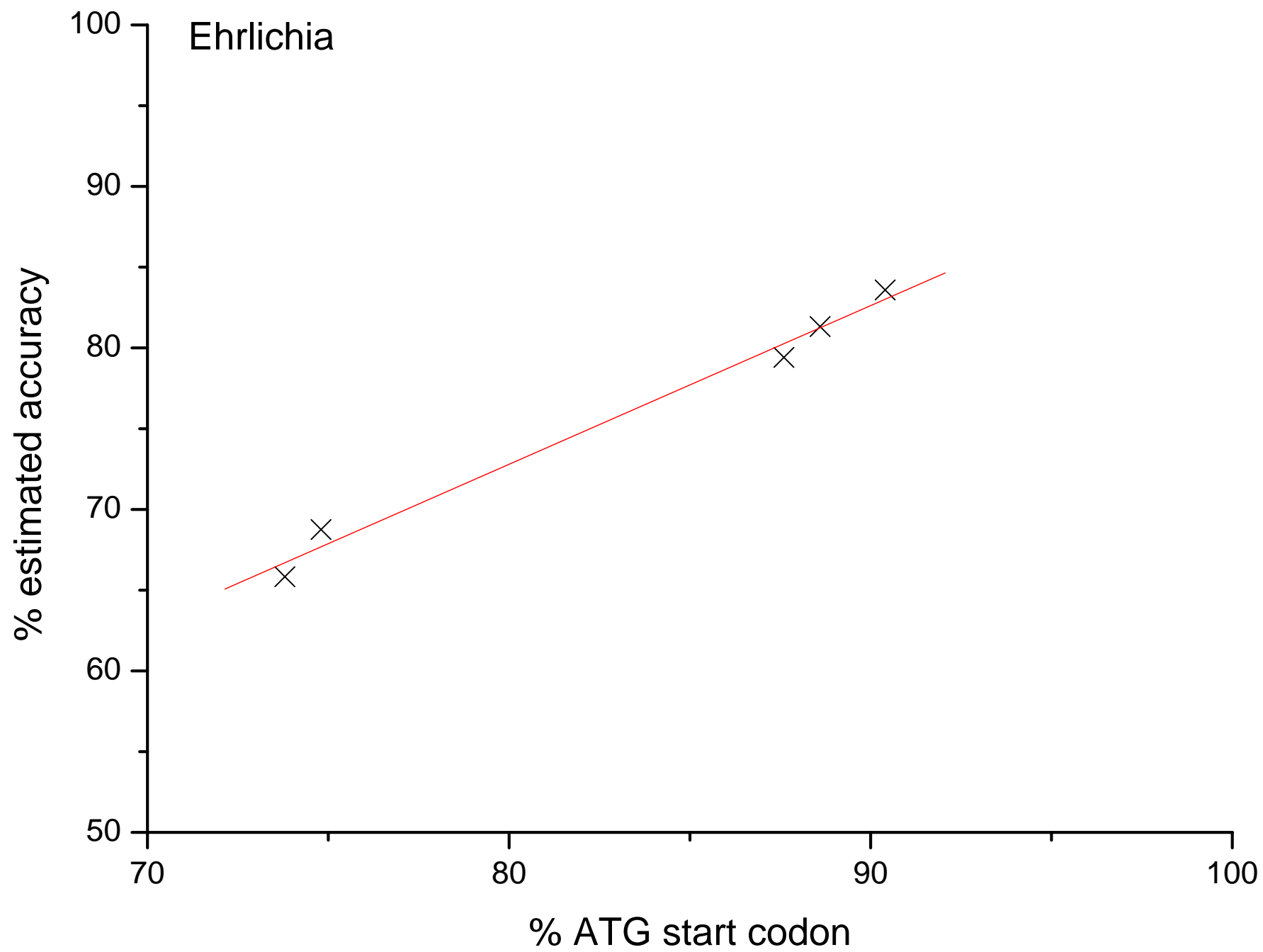

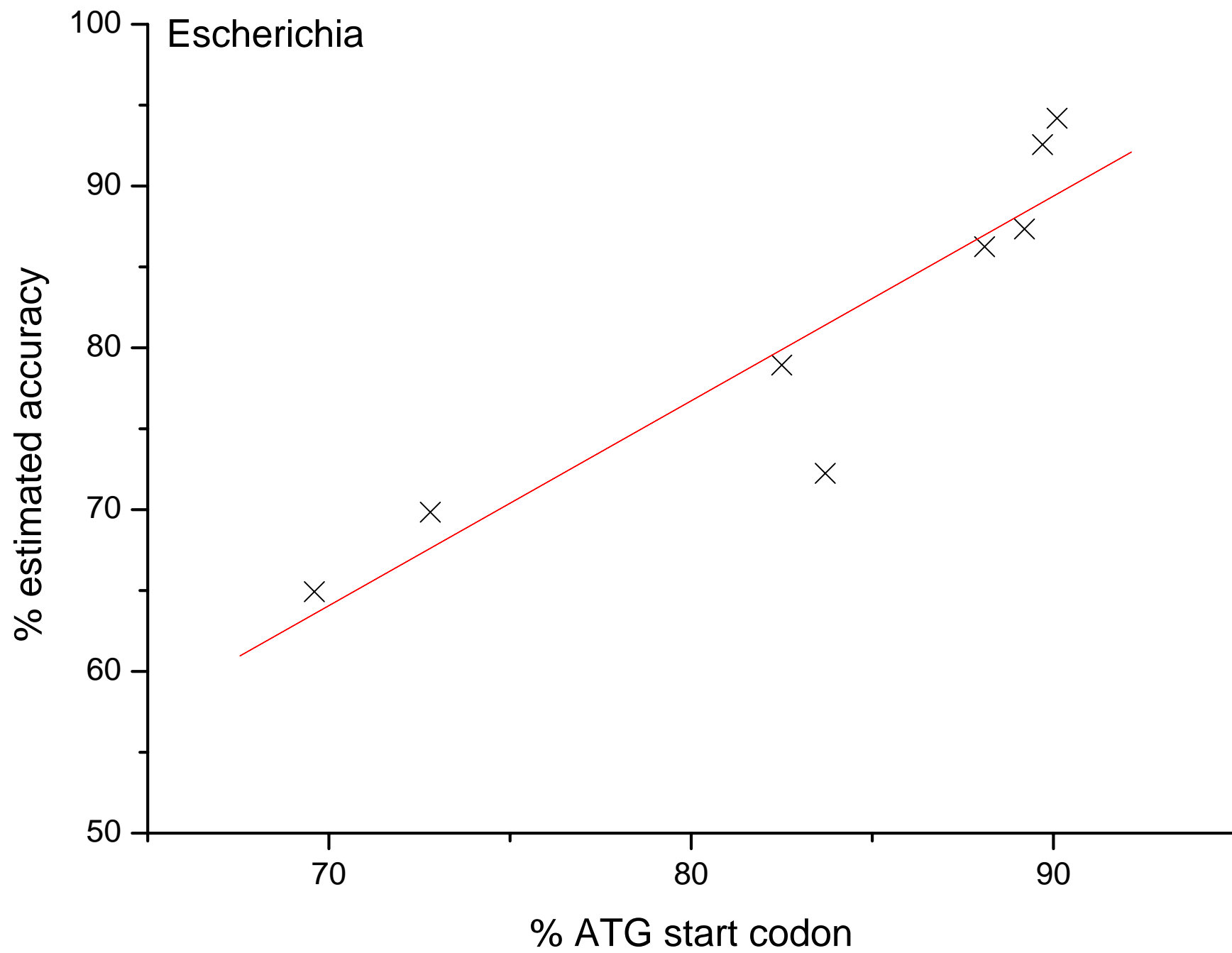

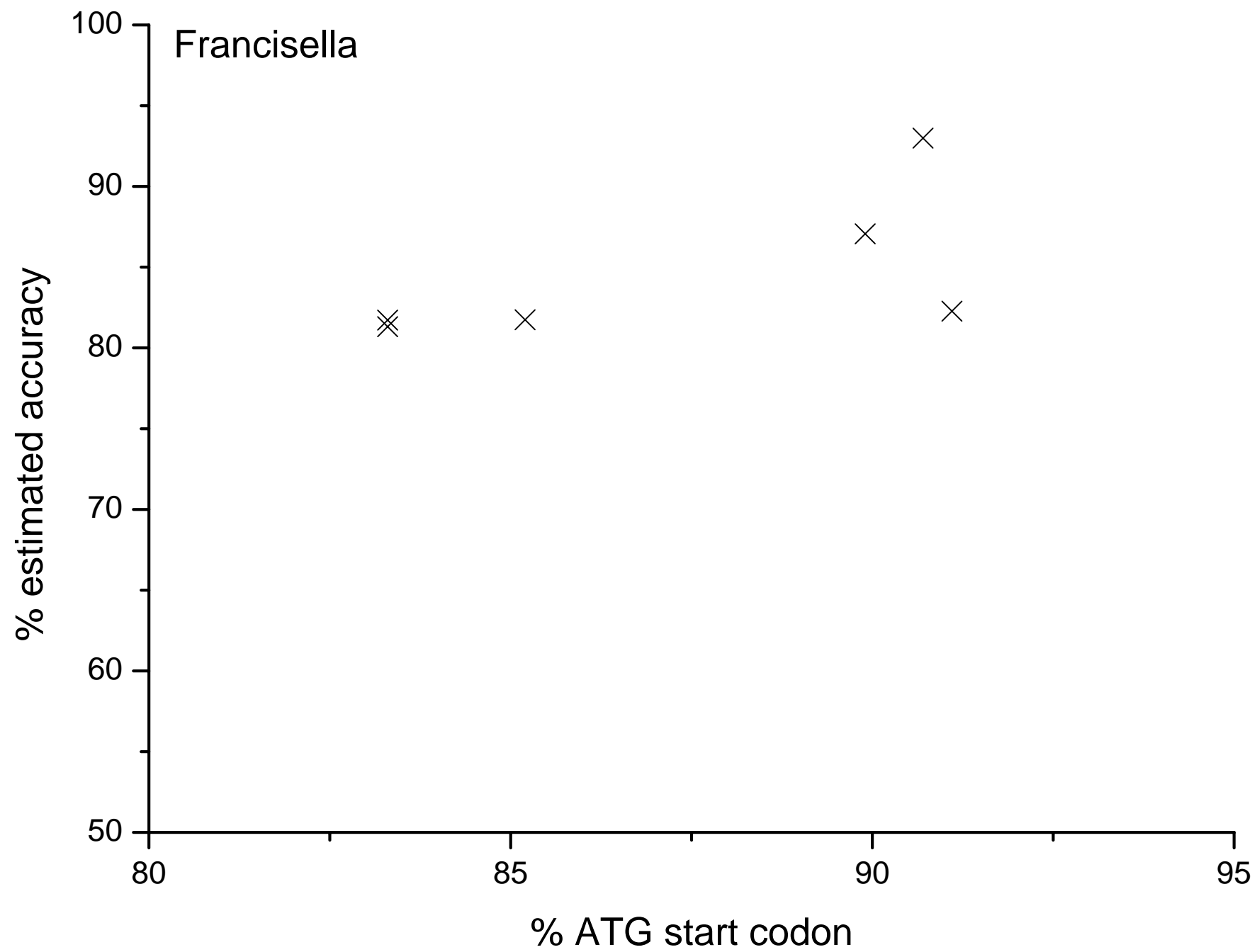

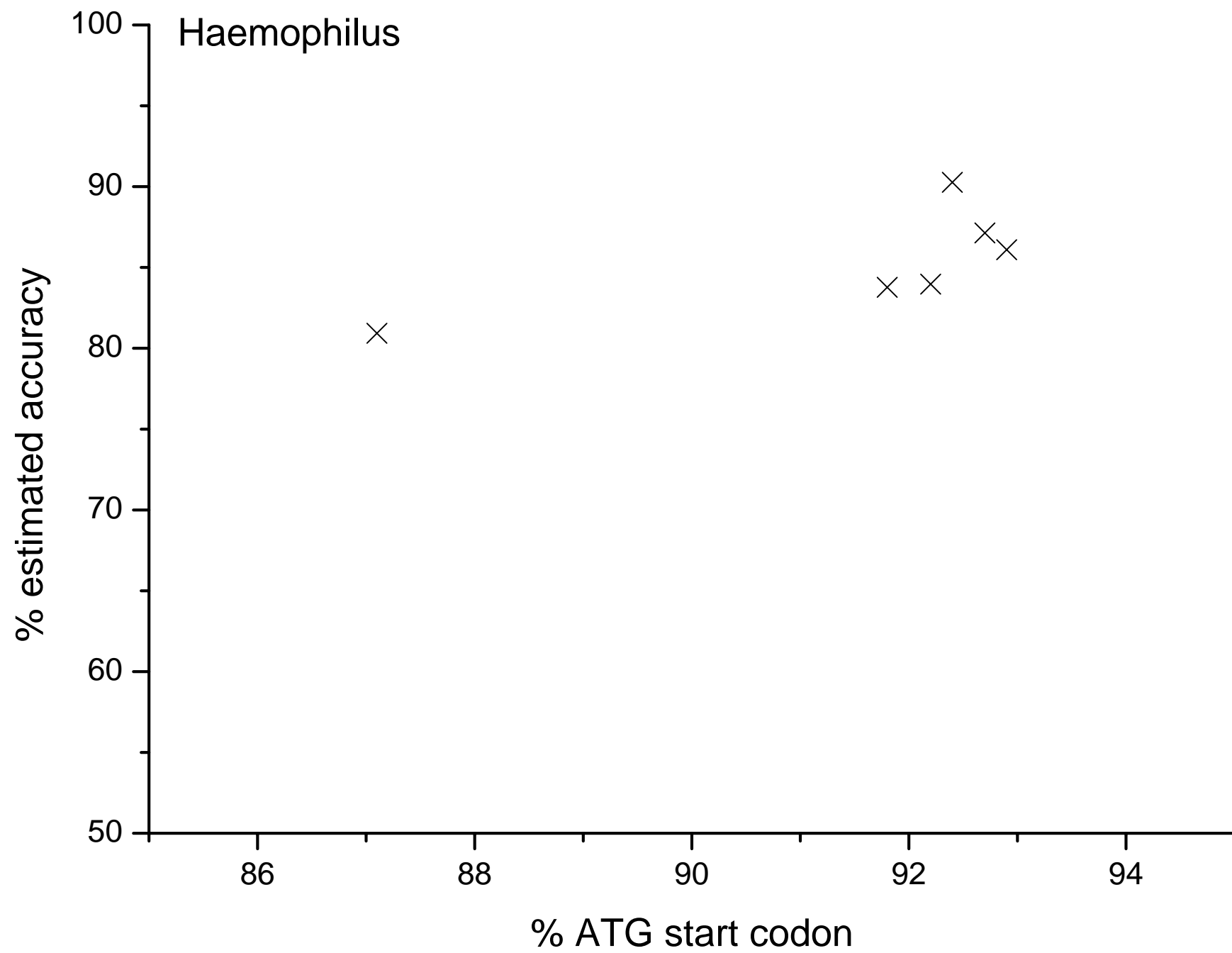

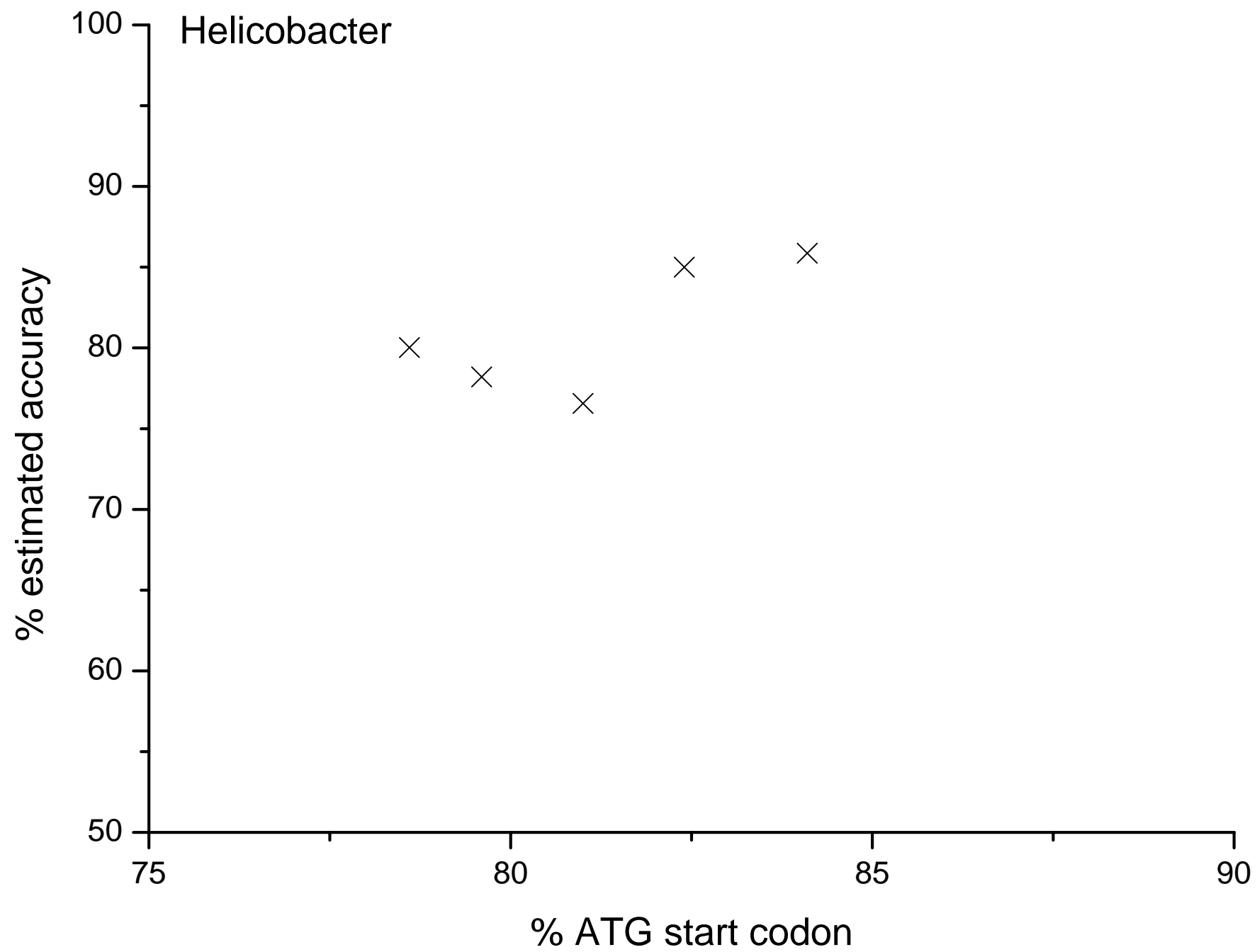

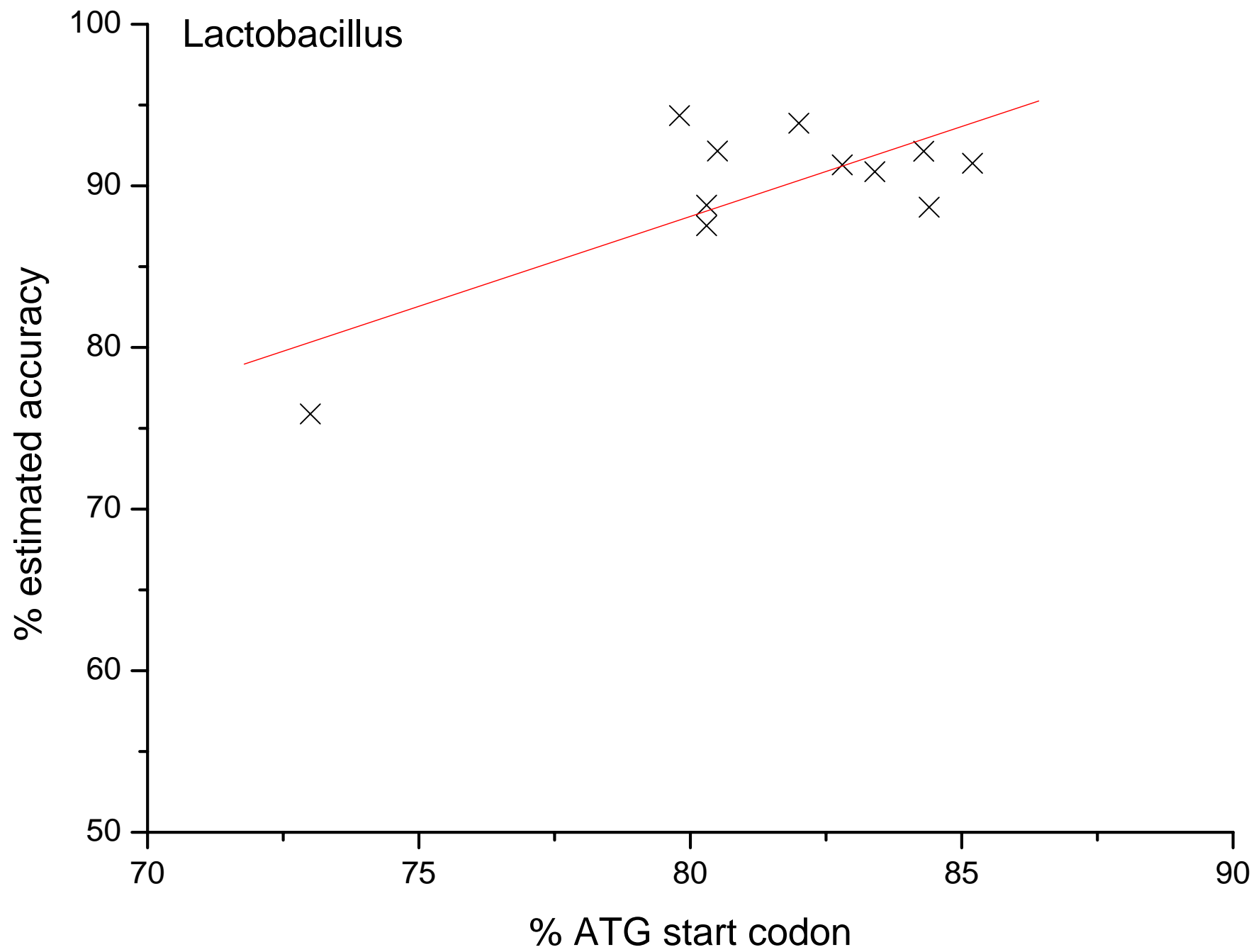

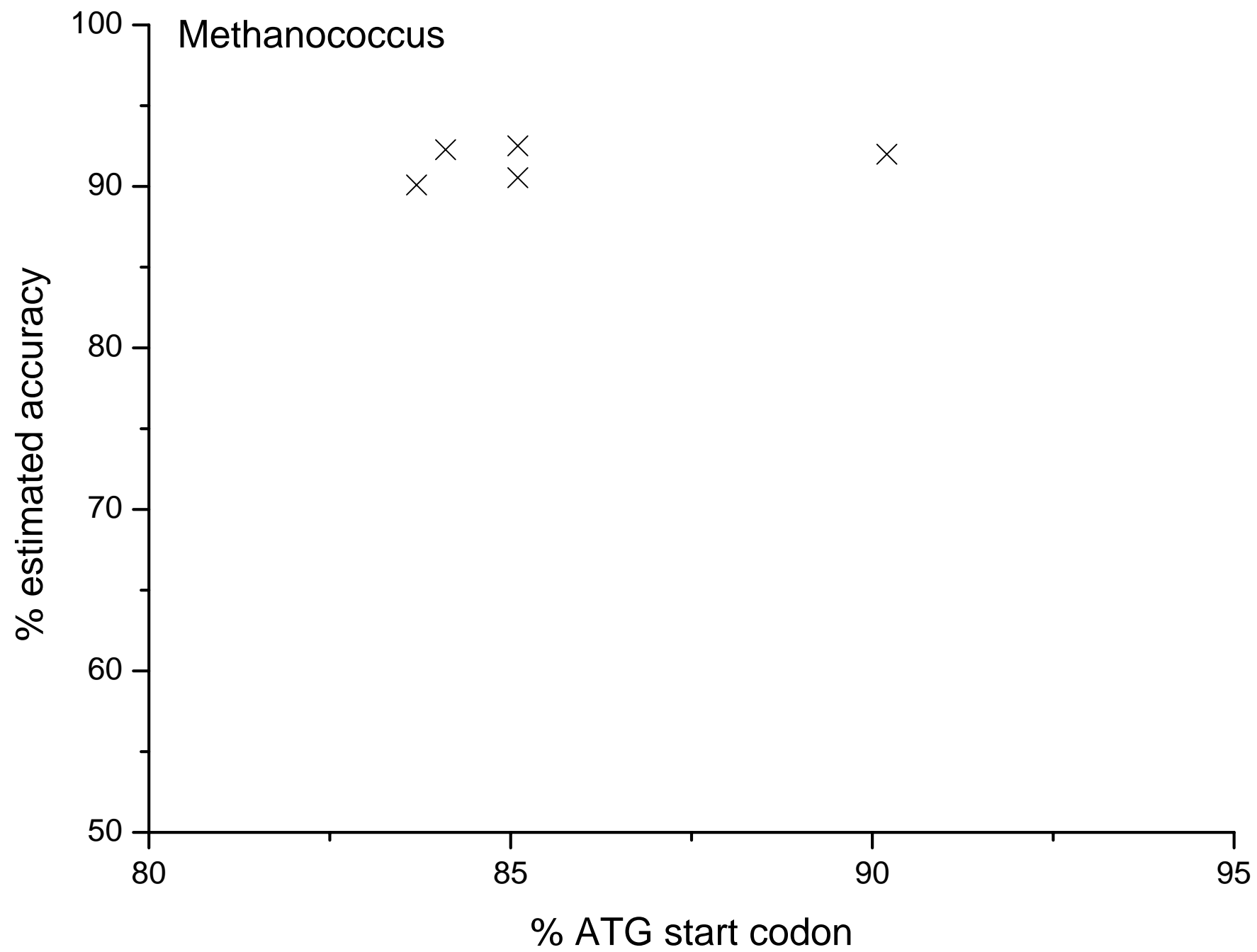

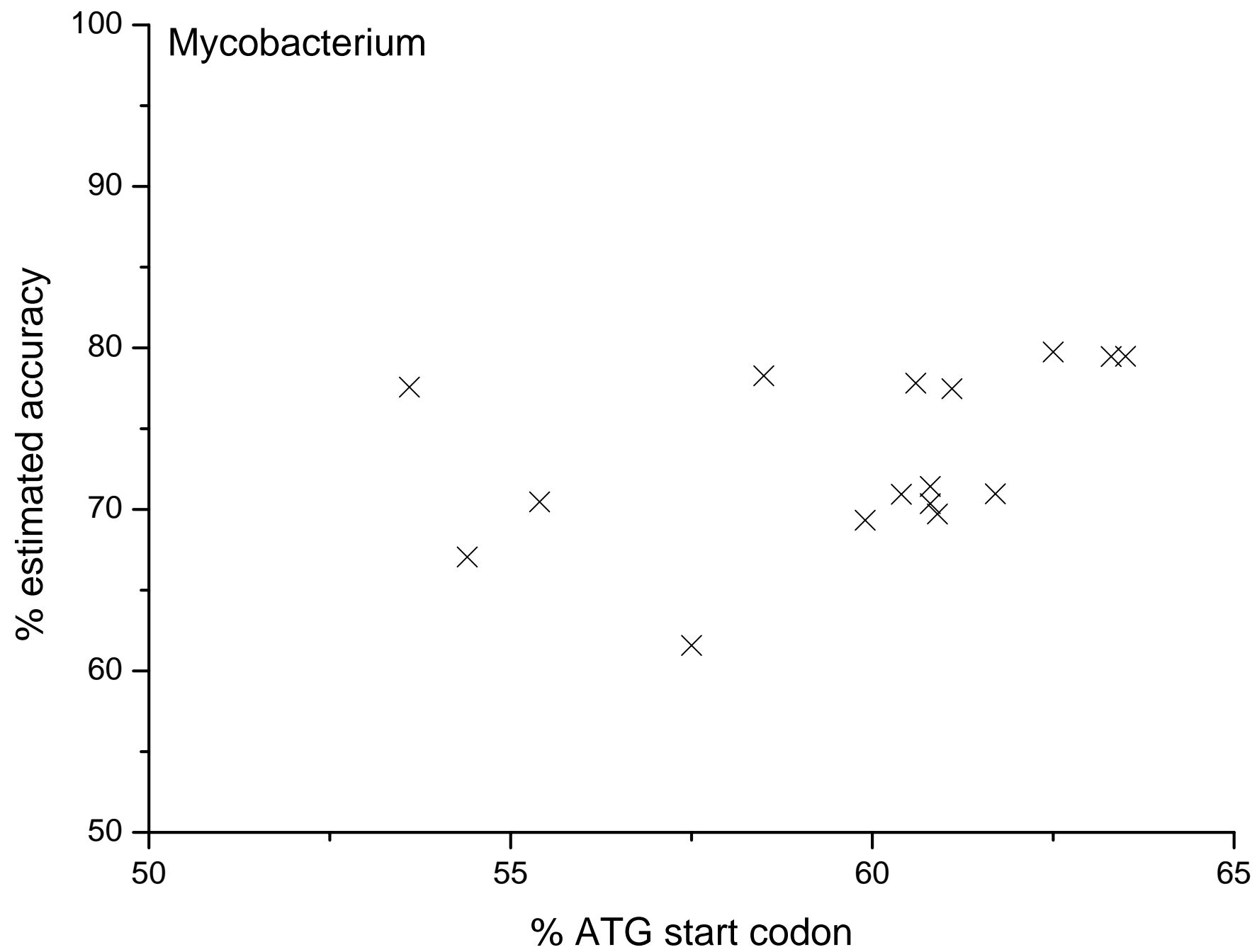

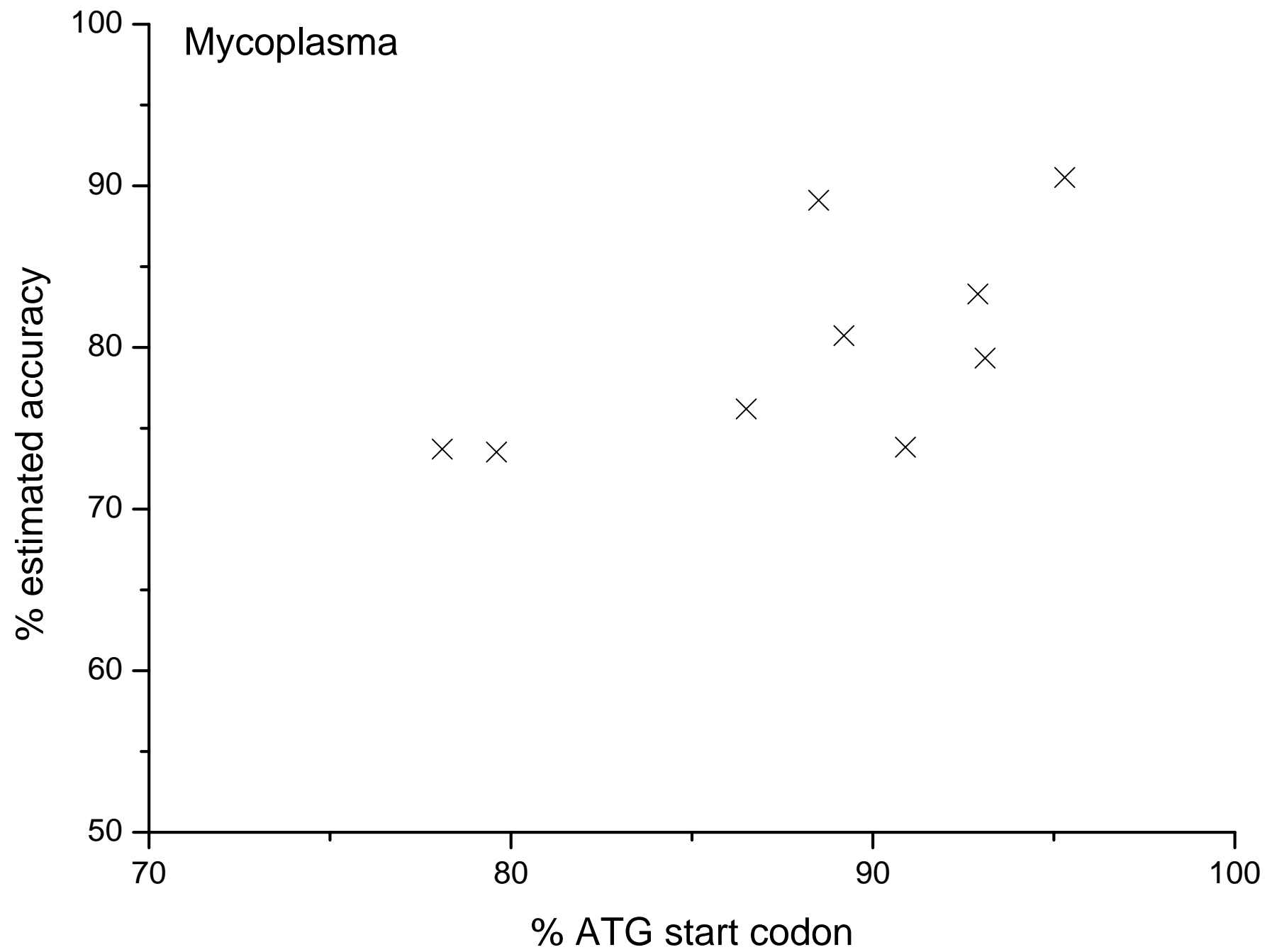

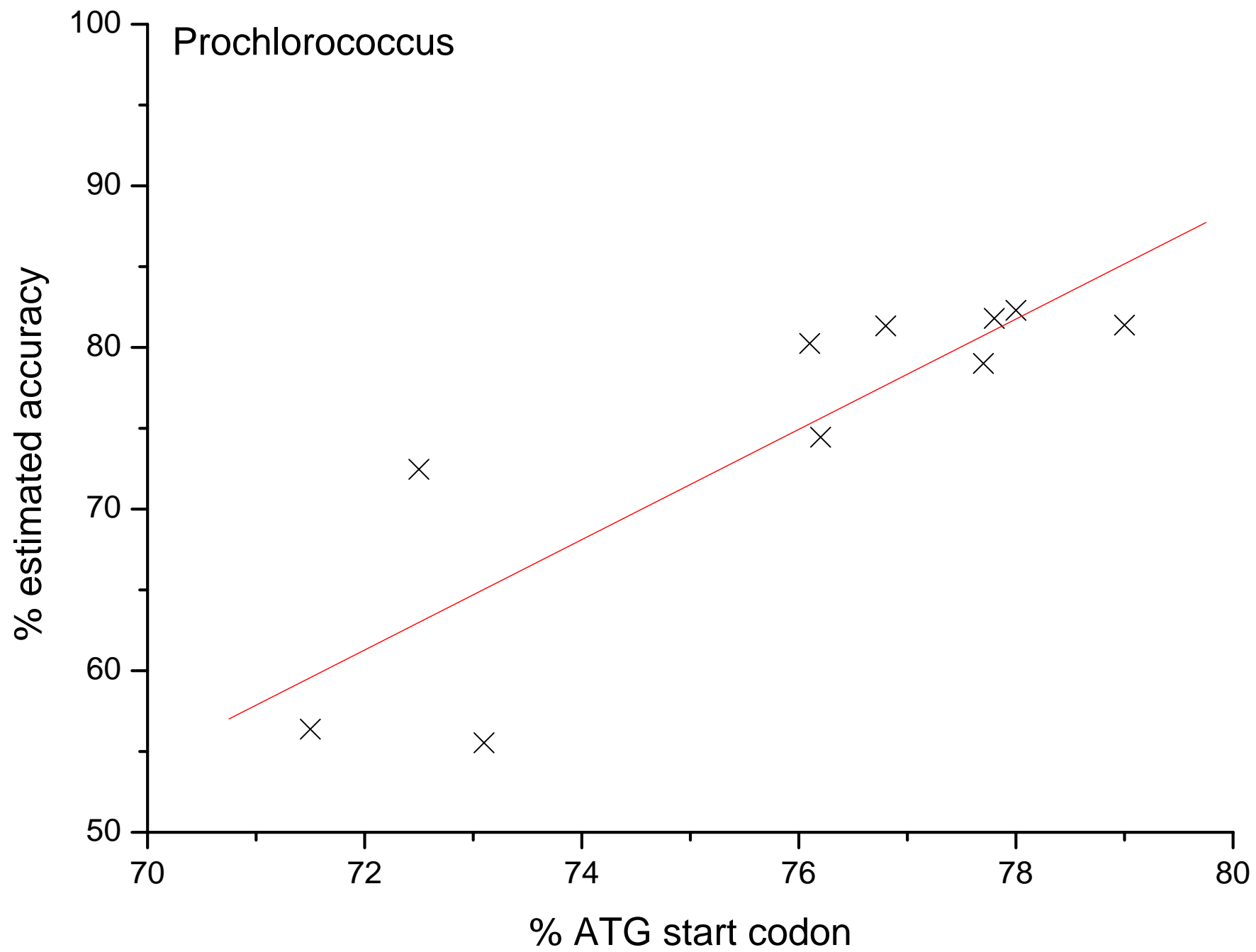

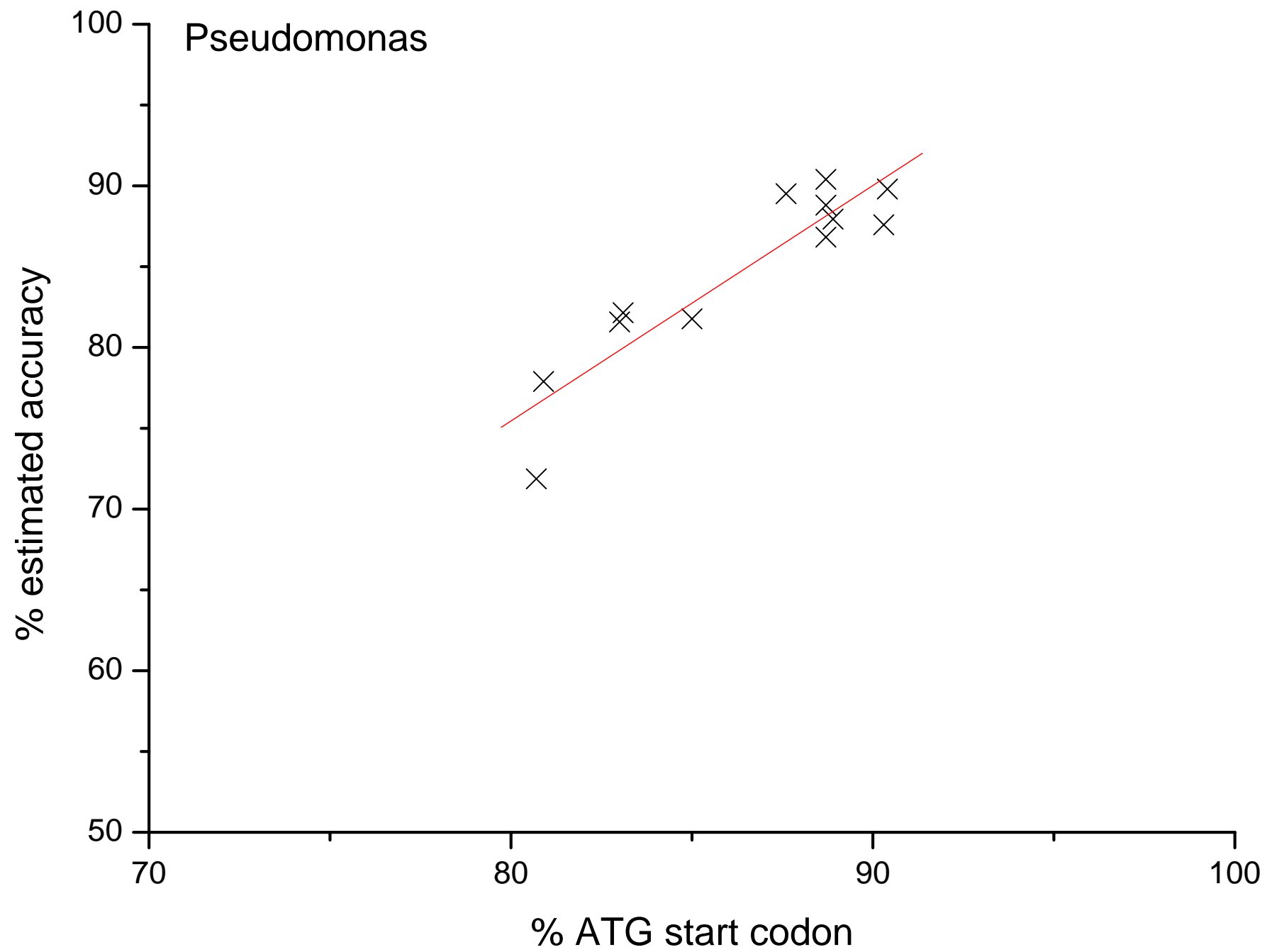

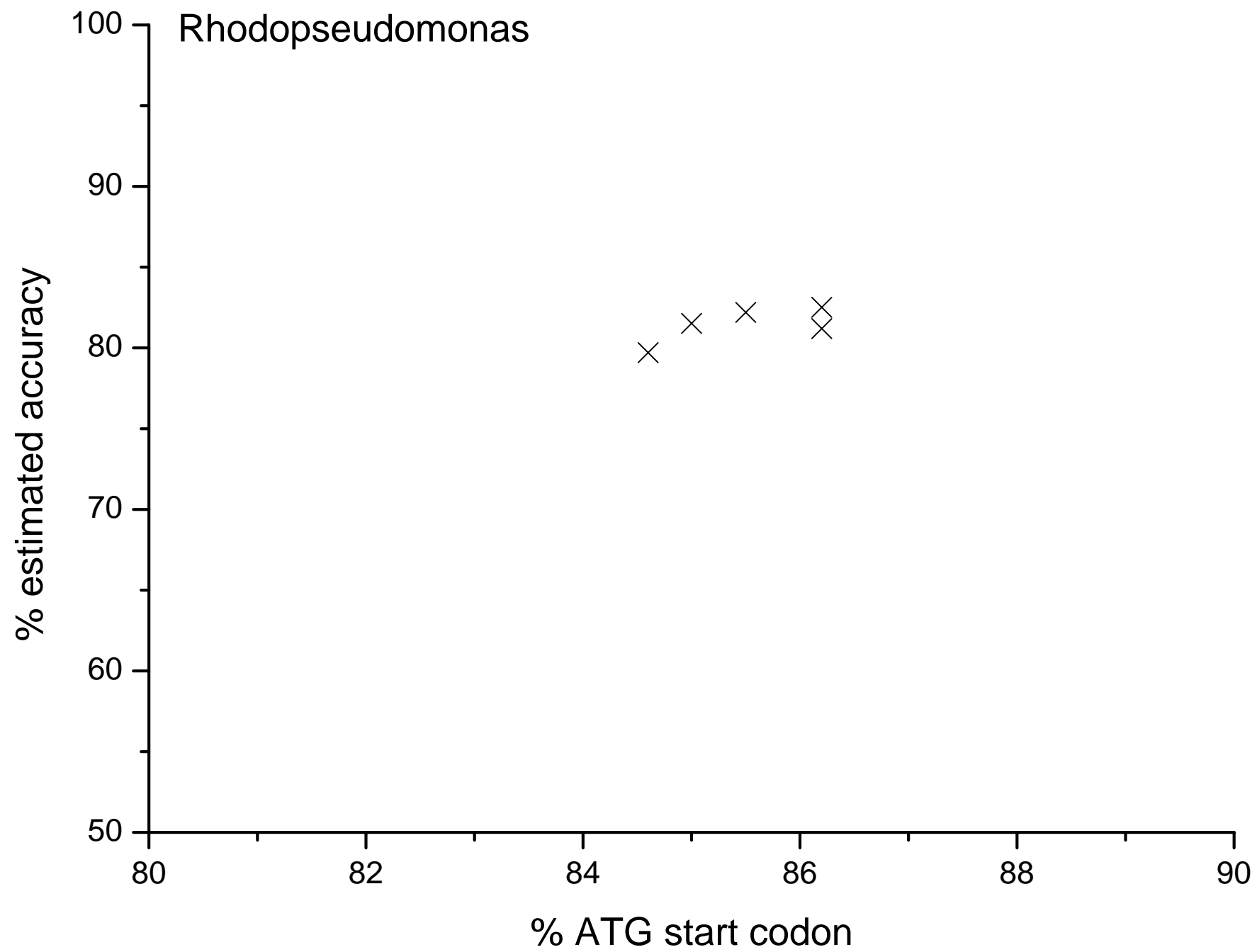

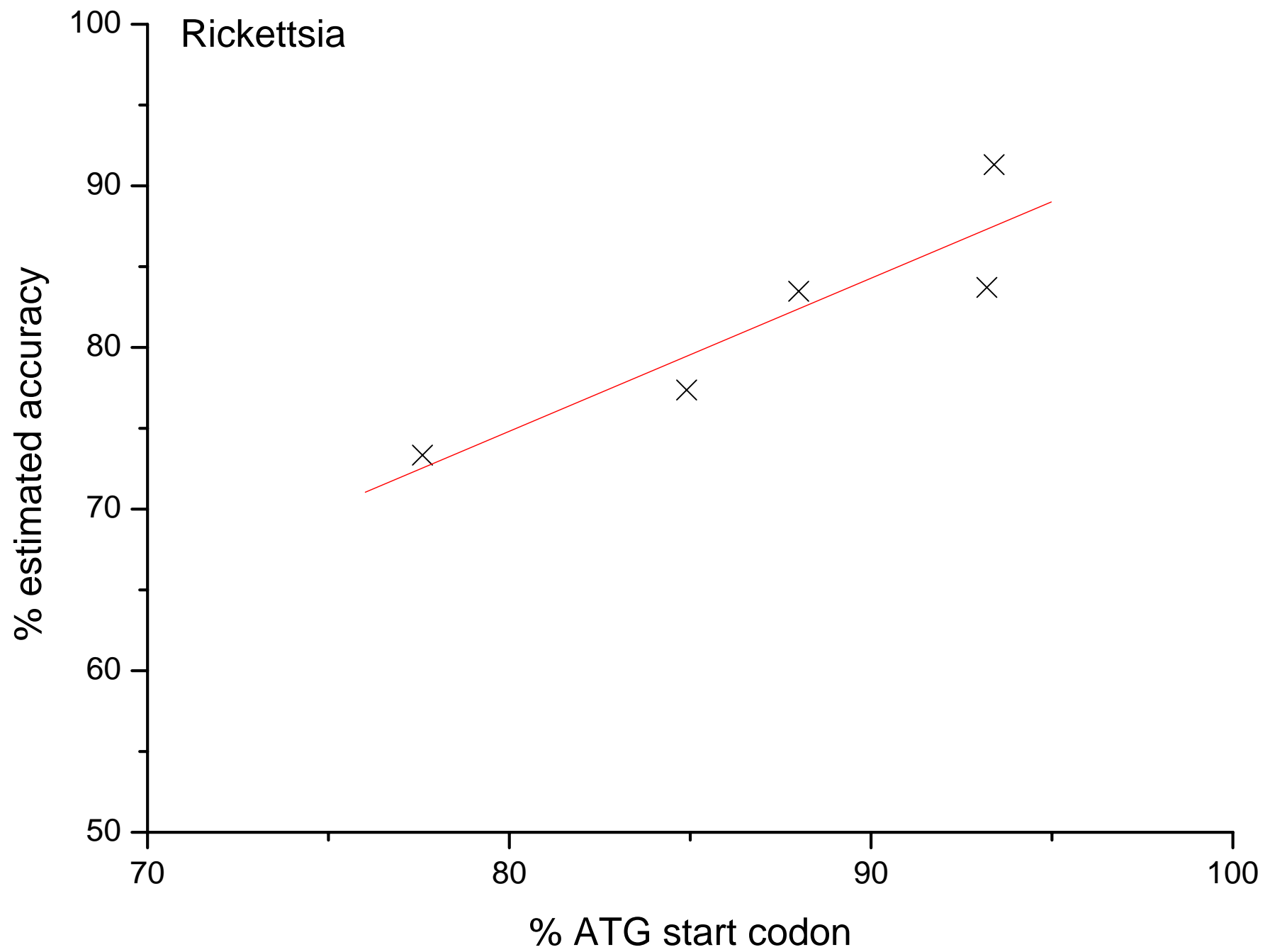

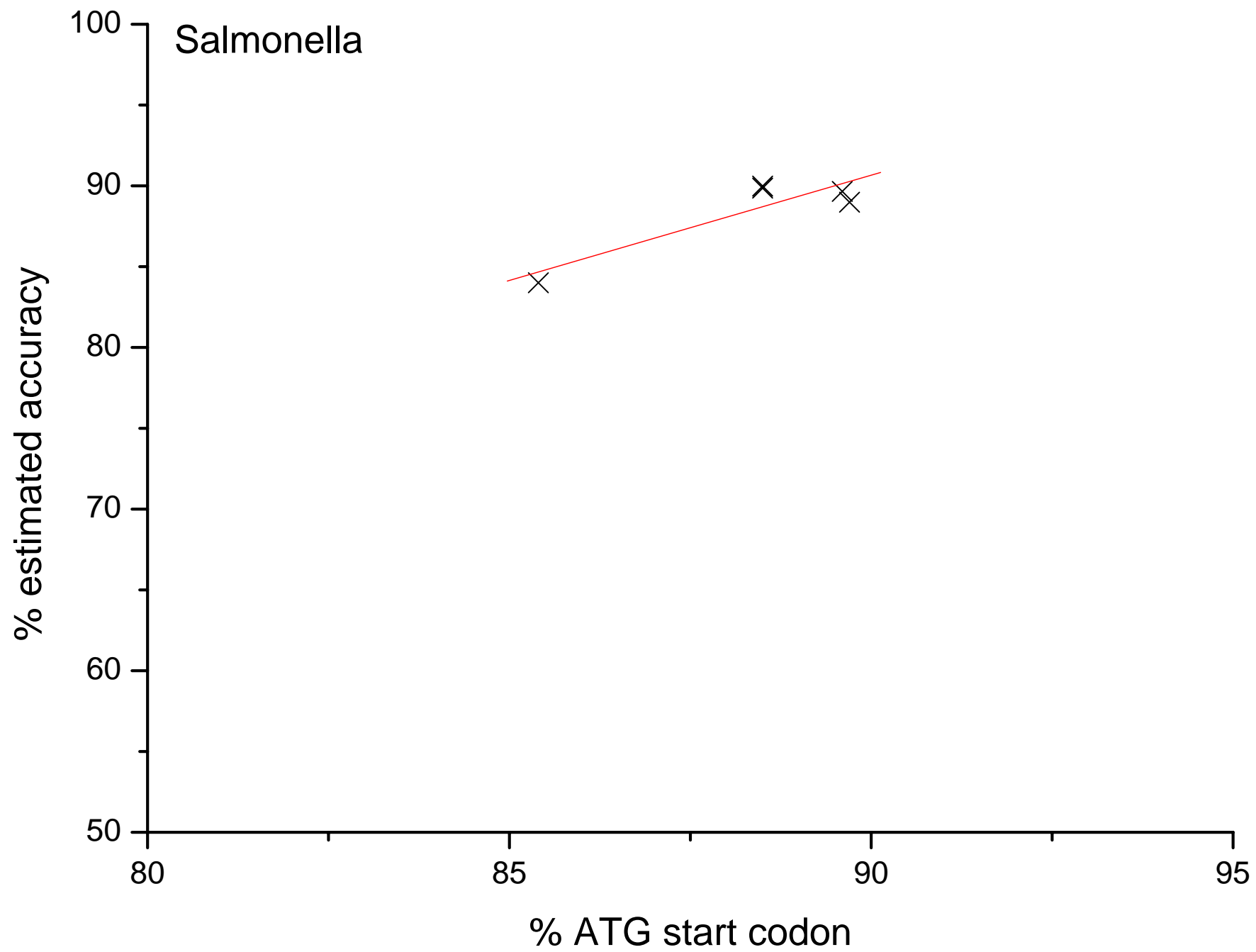

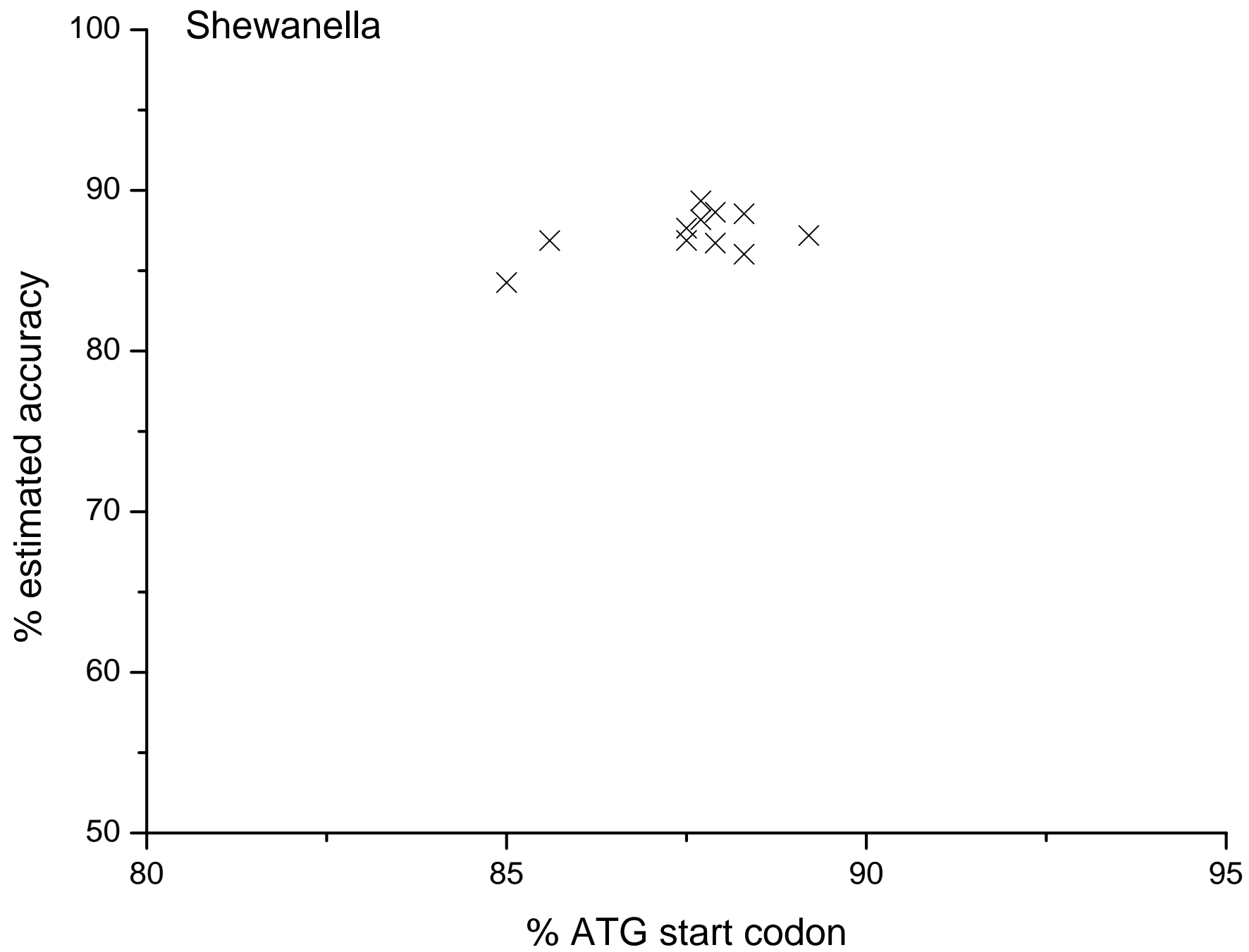

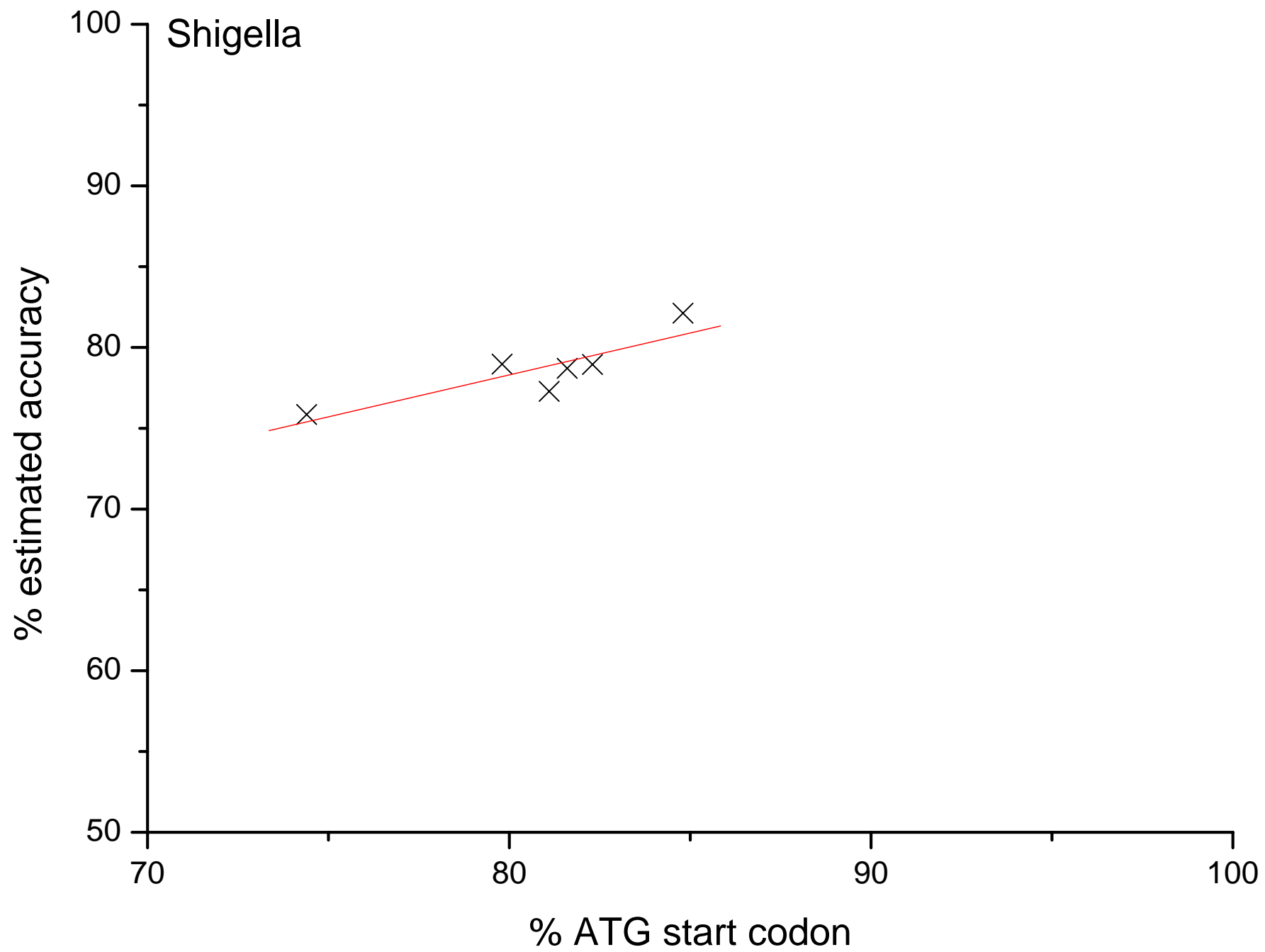

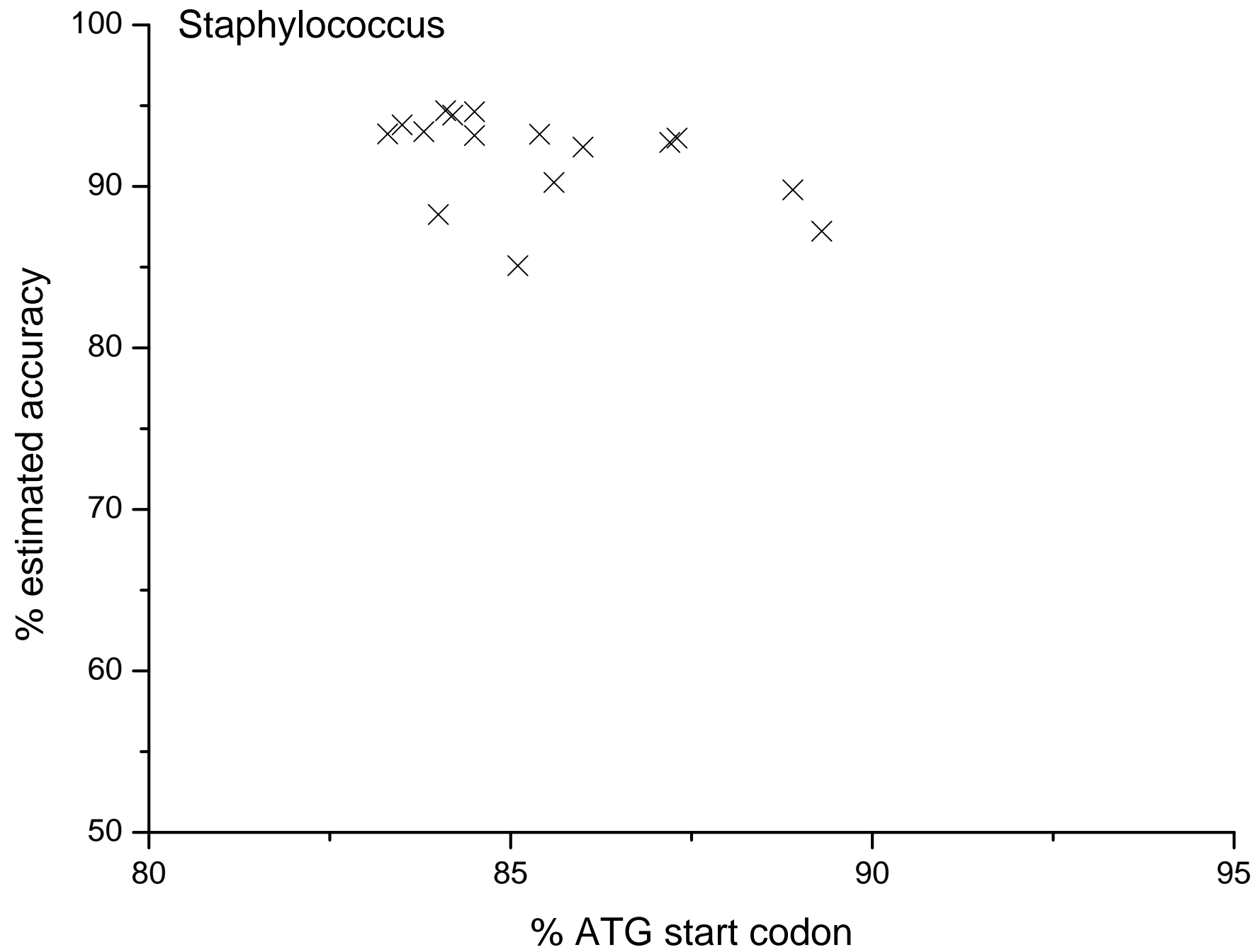

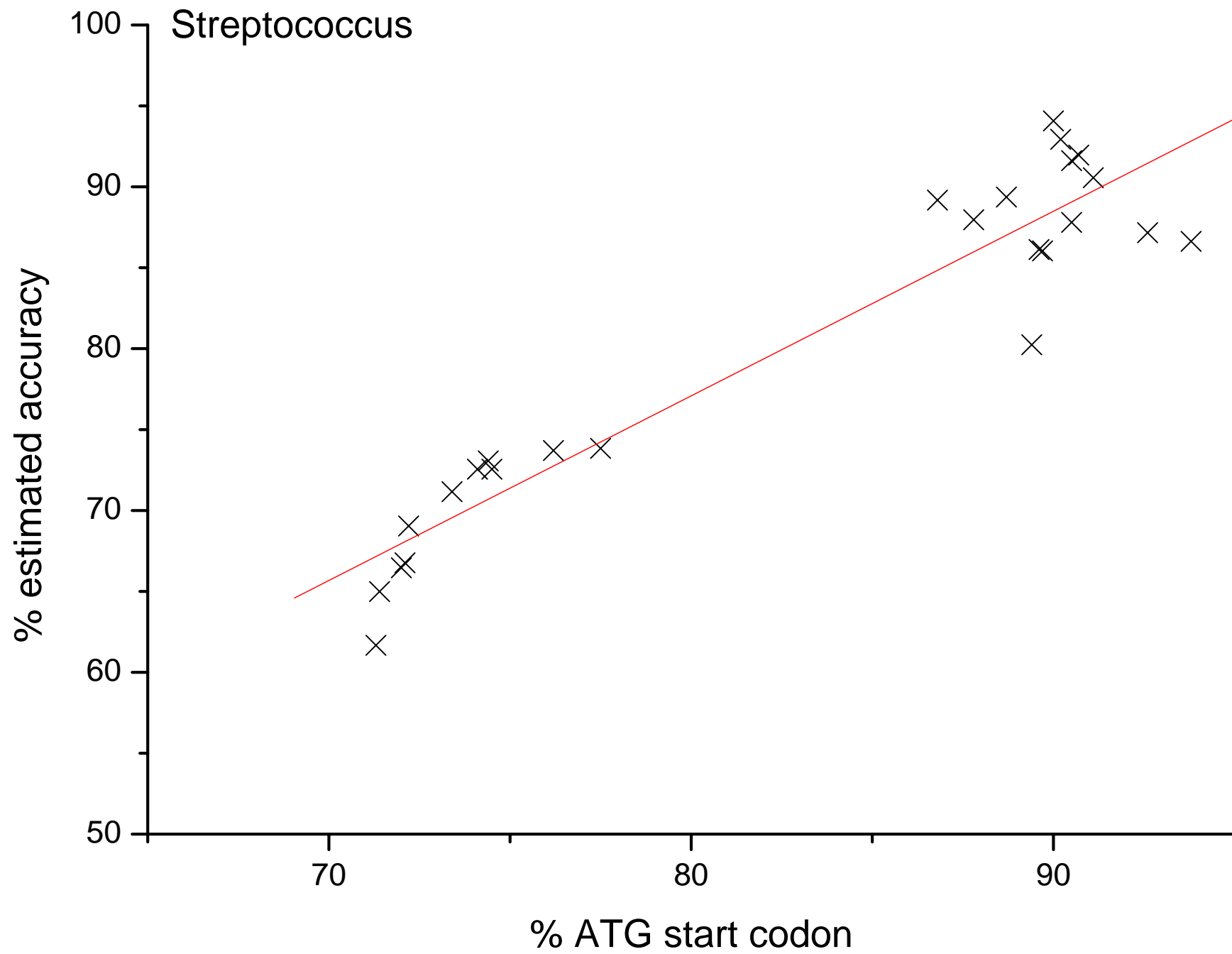

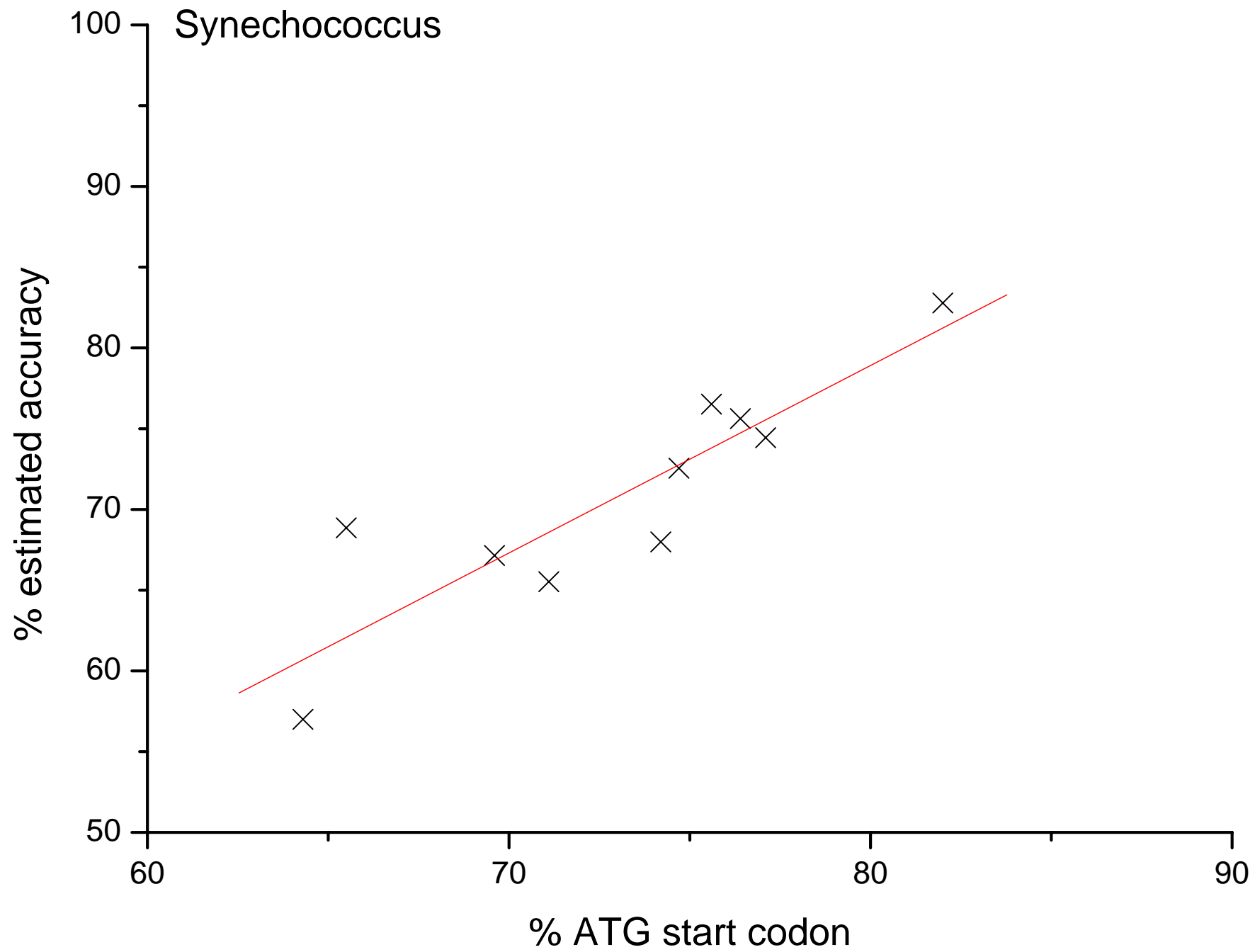

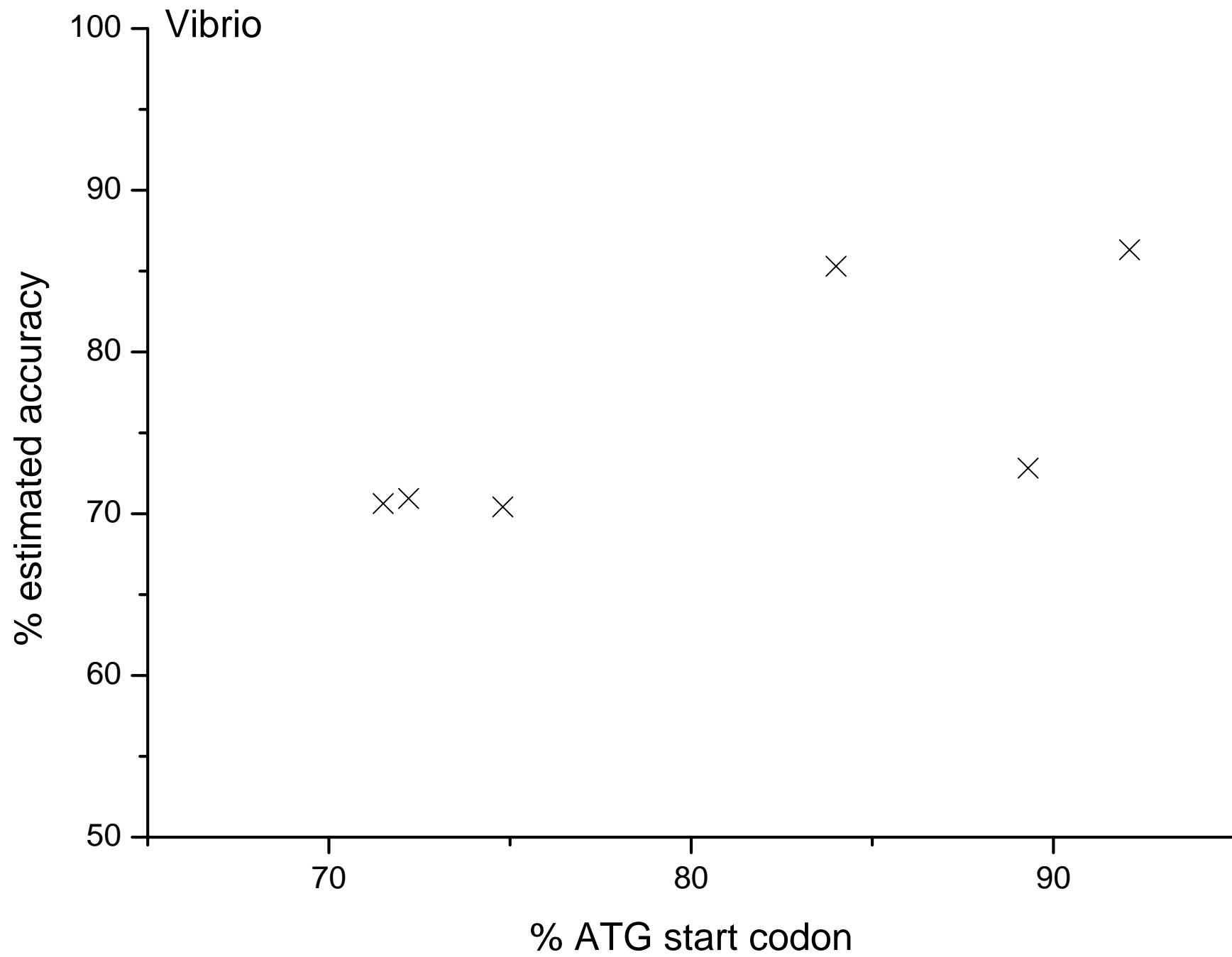

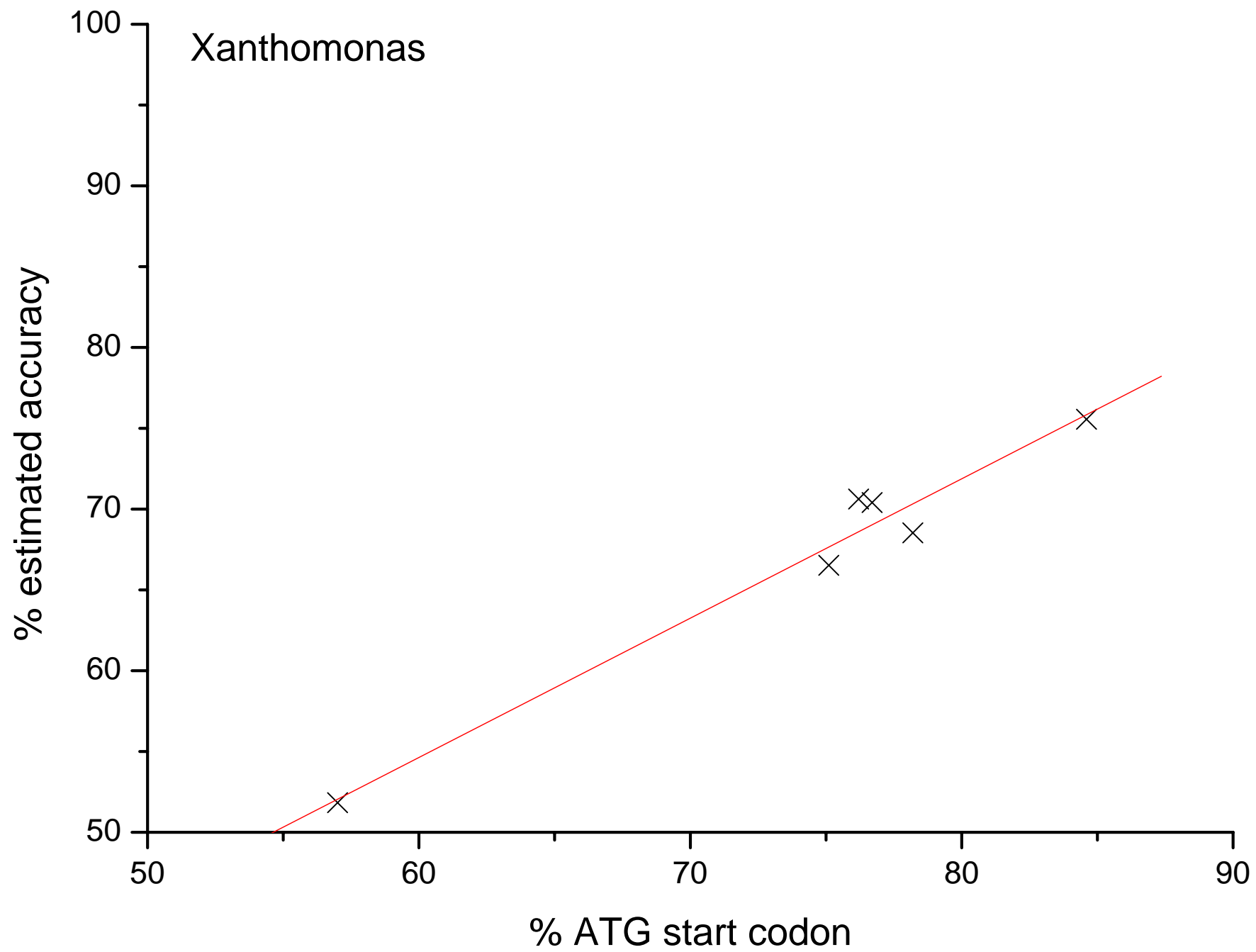

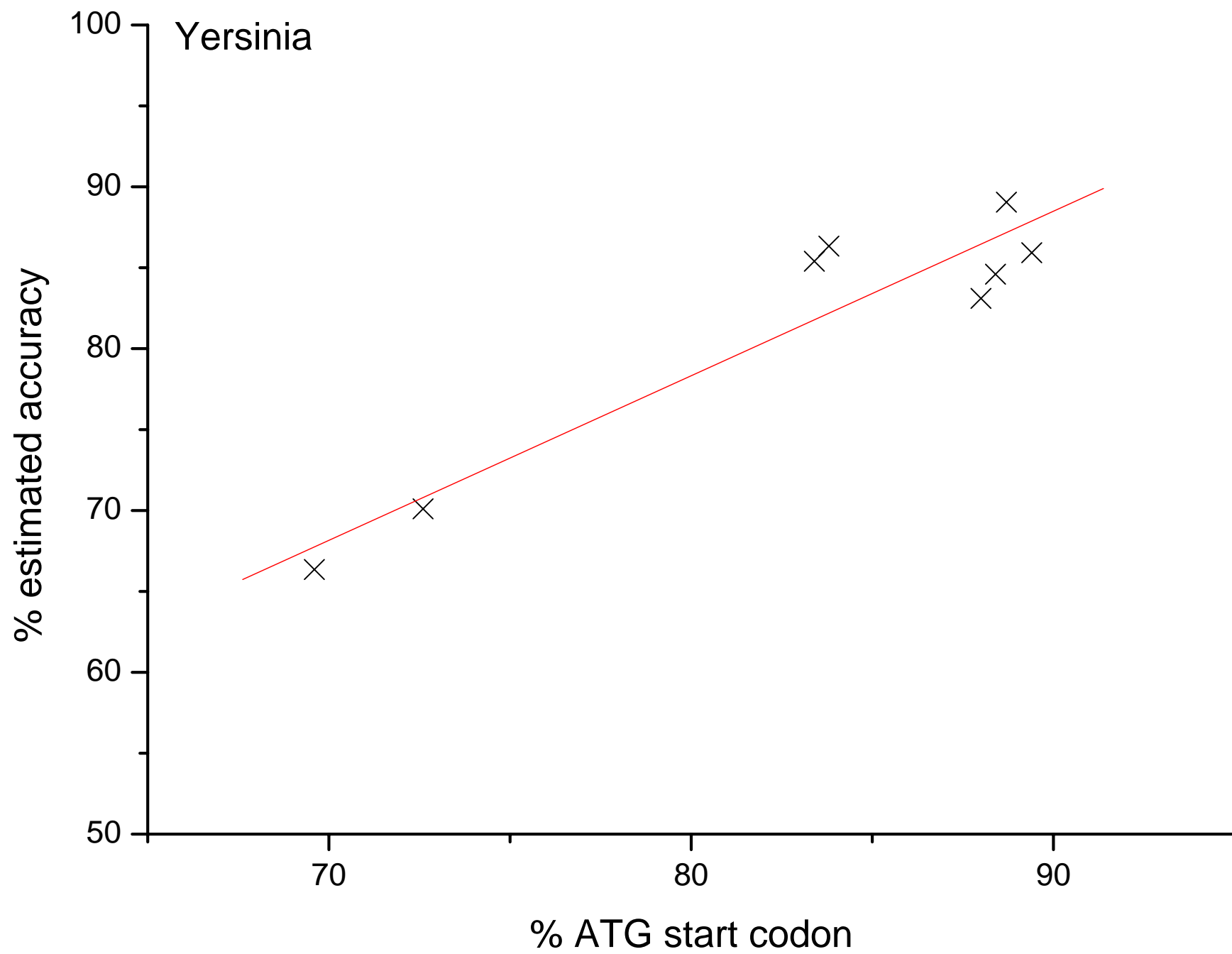

Supplement: Additional file 3 — Correlation between annotation accuracy and ATG start codon usage. A total of 29 genera were selected. The linear fit was applied if the Pearson Correlation is significant at 95% confidence. [file 1471-2105-9-160-S3.pdf]
